# Spectral Vegetation Indices to Track Senescence Dynamics in Diverse Wheat Germplasm

**DOI:** 10.3389/fpls.2019.01749

**Published:** 2020-01-28

**Authors:** Jonas Anderegg, Kang Yu, Helge Aasen, Achim Walter, Frank Liebisch, Andreas Hund

**Affiliations:** ^1^ Crop Science Group, Institute of Agricultural Sciences, ETH Zurich, Zurich, Switzerland; ^2^ Division of Forest, Nature and Landscape, Department of Earth and Environmental Sciences, KU Leuven, Leuven, Belgium

**Keywords:** high-throughput phenotyping, canopy reflectance, hyperspectral remote sensing, field-based phenotyping, feature selection

## Abstract

The ability of a genotype to stay green affects the primary target traits grain yield (GY) and grain protein concentration (GPC) in wheat. High throughput methods to assess senescence dynamics in large field trials will allow for (i) indirect selection in early breeding generations, when yield cannot yet be accurately determined and (ii) mapping of the genomic regions controlling the trait. The aim of this study was to develop a robust method to assess senescence based on hyperspectral canopy reflectance. Measurements were taken in three years throughout the grain filling phase on >300 winter wheat varieties in the spectral range from 350 to 2500 nm using a spectroradiometer. We compared the potential of spectral indices (SI) and full-spectrum models to infer visually observed senescence dynamics from repeated reflectance measurements. Parameters describing the dynamics of senescence were used to predict GY and GPC and a feature selection algorithm was used to identify the most predictive features. The three-band plant senescence reflectance index (PSRI) approximated the visually observed senescence dynamics best, whereas full-spectrum models suffered from a strong year-specificity. Feature selection identified visual scorings as most predictive for GY, but also PSRI ranked among the most predictive features while adding additional spectral features had little effect. Visually scored delayed senescence was positively correlated with GY ranging from r = 0.173 in 2018 to r = 0.365 in 2016. It appears that visual scoring remains the gold standard to quantify leaf senescence in moderately large trials. However, using appropriate phenotyping platforms, the proposed index-based parameterization of the canopy reflectance dynamics offers the critical advantage of upscaling to very large breeding trials.

## Introduction

Maximizing carbon assimilation by a prolonged green leaf area duration after anthesis is a major breeding aim in many crops. This so-called stay green (Thomas and Smart, 1993) has been linked to increased grain yield (GY) in several crops (*reviewed by* Gregersen et al., 2013). Stay green results from a delayed onset of senescence and/or a reduction in the rate of the process (Gregersen et al., 2013). The benefit of such an extended period of functional stay green, i.e. a prolonged photosynthetic activity, has been particularly well documented in maize and sorghum (Rajcan and Tollenaar, 1999; Borrell et al., 2000).

In wheat, potential GY is currently viewed as being predominately limited by sink strength, i.e. the number of grains available for grain filling, which is largely determined up until and including a short period after anthesis (*reviewed by* Borrás et al., 2004; Fischer, 2008; Distelfeld et al., 2014). However, several studies have reported positive correlations between delayed senescence and GY, particularly under stress conditions (Verma et al., 2004; Christopher et al., 2008; Bogard et al., 2011; Lopes and Reynolds, 2012; Christopher et al., 2014; Christopher et al., 2016; Montazeaud et al., 2016). Where plants are exposed to severe stress, the stay-green phenotype may be interpreted as the avoidance of premature senescence, which could result in source limitation, i.e. a lack of carbohydrates delivered to the developing grains (Borrás et al., 2004). Fine-tuning senescence dynamics has therefore been proposed as a promising selection criterion in wheat breeding particularly under the scenario of an increased frequency of weather extremes, such as heat and drought.

Optimizing senescence dynamics requires intense field testing for at least two reasons: (i) senescence *per se* is known to underlie complex genetic and environmental control (*reviewed by* Lim et al., 2007), typically resulting in moderate to low heritability across environments (*e.g.* Lopes and Reynolds, 2012; Crain et al., 2017) and (ii) effects of altered senescence dynamics on key primary traits, such as GY and grain protein concentration (GPC), often depend on the environment (Bogard et al., 2011; Lopes and Reynolds, 2012). For example, negative relationships between GY and stay-green have also been reported, especially in the absence of water- or nitrogen-limiting conditions (Jiang et al., 2004; Derkx et al., 2012; Naruoka et al., 2012; Kipp et al., 2014). A delayed or slow senescence has also been linked to a reduced efficiency of remobilization, with adverse effects on harvest index (Gong et al., 2005; Yang and Zhang, 2006), nitrogen use efficiency and GPC (Gregersen et al., 2008; Gaju et al., 2014). GPC is a key quality parameter in bread wheat, which may be additionally lowered *via* a dilution effect if the increased post-anthesis C-compound synthesis of stay-green cultivars is not paralleled by an increased uptake and transfer of nitrogen to the developing grains (Bogard et al., 2010; Cormier et al., 2016). Thus, in order to exploit variation in senescence dynamics for the improvement of bread wheat, a better understanding of environmental, genetic, and physiological determinants of senescence dynamics *per se* as well as of the effects of senescence dynamics on GY and GPC in contrasting environments is required. Traditional phenotyping methods, such as visual senescence inspection (*e.g.* Bogard et al., 2011) or SPAD meter measurements (*e.g.* Xie et al., 2016) do not provide the necessary throughput to assess a dynamic trait for large numbers of genotypes at high temporal resolution and in contrasting environments.

Regular ground-based normalized difference vegetation index (NDVI) measurements obtained from an active spectral GreenSeeker sensor (NTech Industries, Ukiah, CA, USA) have shown significant potential for the rapid identification of variation in senescence patterns among wheat genotypes (Lopes and Reynolds, 2012; Christopher et al., 2014; Montazeaud et al., 2016). However, the use of a single and relatively unspecific spectral index is likely to entail some important limitations. During senescence, wheat canopies undergo a sequence of profound biochemical and biophysical changes. These changes in part temporally overlap, and their effects on the reflectance spectrum of the canopy are, therefore, confounded. In this context, to the best of our knowledge, the NDVI has been used primarily as a generic indicator of canopy greenness or green biomass and has not been thoroughly validated as a tool to track canopy senescence in wheat. Gitelson and Merzlyak (1994) demonstrated the insensitivity of the NDVI to physiological changes occurring during early senescence at the leaf scale. At the canopy scale, the NDVI is often saturated in dense canopies as can be observed for wheat stands under favorable conditions (Asrar et al., 1984; Gu et al., 2013). This is likely to limit the sensitivity and precision of the NDVI in detecting early senescence at the canopy scale. Using passive sensors with a high spectral resolution, more specific narrow-band spectral indices (SI) or full-spectrum analysis can be deployed to reduce the effect of canopy structure and other confounding factors on the assessment of biochemical or physiological traits of interest (*e.g.* Haboudane et al., 2002; Chen et al., 2010; Li et al., 2014). For example, the plant senescence reflectance index (PSRI) developed by Merzlyak et al. (1999) can be used to measure leaf and fruit senescence. It is based on the chlorophyll/carotenoid ratio which undergoes major changes as a consequence of differential breakdown rates of these pigments during early senescence, offering advantages over the NDVI (Sanger, 1971; Fischer and Feller, 1994; Merzlyak et al., 1999). Similarly, Kipp et al. (2014) were able to estimate greenness of flag leaves and onset of flag leaf senescence in wheat using ground-based hyperspectral canopy reflectance measurements in combination with full-spectrum models, while no stable relationships were found for the NDVI.

An additional advantage of hyperspectral reflectance measurements as compared to single SI measurements could consist in the opportunity to track multiple processes simultaneously. For example, during late development, green leaf area, pigment composition and total content, nitrogen distribution and water content of the canopy change dramatically. Visual senescence scorings mainly capture changes in pigment composition and content, but largely disregard other canopy characteristics, potentially resulting in a loss of breeding-relevant information. For example, the dynamics of nitrogen remobilization after flowering has been identified as a key determinant of GPC in wheat (*reviewed by* Kong et al., 2016). In contrast to visual scorings, all of the aforementioned traits have been shown to be amenable to assessment using hyperspectral measurements provided sufficient variability exists (Haboudane et al., 2002; Li F. et al., 2014; Li X. et al., 2014; Becker and Schmidhalter, 2017). In a breeding context, variability for a trait of interest is typically low, and differences in morphology and canopy structure among genotypes are thus likely to mask their effects on spectral reflectance at a specific point in time. However, assessments of relative changes over time could reveal differences in trait dynamics, which can be analyzed at the level of genotypes or experimental plots. Thus, we hypothesized that capturing the dynamics of such traits using repeated reflectance measurements during late development could complement a precise representation of canopy greenness. The objective of the present study was two-fold: First, we aimed to develop a high-throughput method based on spectral reflectance to track visually observed senescence dynamics in a large population of morphologically diverse wheat genotypes. Second, we aimed to establish whether the resulting representation of canopy greenness decay could be complemented with additional information (*e.g.* relating to pigment, nitrogen or water content of the canopy) derived from repeated hyperspectral reflectance measurements.

## Materials and Methods

### Plant Materials, Experimental Design, and Meteorological Data

A field experiment was conducted in the field phenotyping platform (Kirchgessner et al., 2017) at the ETH Research Station for Plant Sciences Lindau-Eschikon, Switzerland (47.449N, 8.682E, 520 m a.s.l.; soil type: eutric cambisol) in the wheat growing seasons of 2016 to 2018. In each year 300 cultivars comprised in the GABI wheat panel (Kollers et al., 2013) obtained from the Leibniz Institute of Plant Genetics and Crop Plant Research (IPK) were used, which were complemented with important Swiss cultivars for a total of 335 cultivars in 2016 and a total of 352 cultivars in 2017 and 2018. The cultivars were grown in plots of 1 m × 1.4 m size. The designs were generated using the R package DiGGer (Coombes, 2009; http://nswdpibiom.org/austatgen/software). The plots were arranged in a two dimensional incomplete block design with checks. The test varieties were randomized in two complete replications (one per lot). Within each replication, these test varieties were allocated to incomplete row blocks of size one (one row per block) and incomplete range blocks of size six (six ranges per block). The check varieties were distributed as follows: In 2016, wheat cultivar CH CLARO was used as a check variety at 21 evenly distributed locations in each replicate leading to a total of 42 checks per design. In 2017 and 2018 the three Swiss cultivars CH CLARO, SURETTA and NARA (DSP, Delley, Switzerland) were allocated to nine complete blocks spanning seven rows by six ranges each, summing up to a total of 54 checks per design. In all cases, at least one check was present per row and column of the design. Crop husbandry was performed according to local agricultural practice. The experiments were sown with a sowing density of 400 plants m^−2^ on Oct 13, 2015, on Nov 1, 2016, and on Oct 18, 2017, respectively. Temperature data was retrieved from an on-site weather station. Rainfall data was obtained from a nearby weather station of the federal Swiss meteorological network Agrometeo (www.agrometeo.ch) located at ca. 250 m distance to the field trial. The temperature data was used to calculate growing degree-days (GDD) following

$$T{\text{mean}}_{d}=\frac{\sum^{}\frac{\text{max}T_{\text{d}.\text{h}}+\text{min}T_{\text{d},\text{h}}}{2}-\text{base}T}{24}$$

$$\mathrm{GDD}=\sum_{d=1}^{n}T{\text{mean}}_{d}\;$$

where *T*mean_d_ is the mean temperature for day d after heading, max*T*_*d*,*h*_ and min*T*_*d*,*h*_are hourly maximum and minimum temperatures for day d and base*T* is the base temperature, set to 0°C.

### Phenology and Agronomic Data

**Figure 1:**
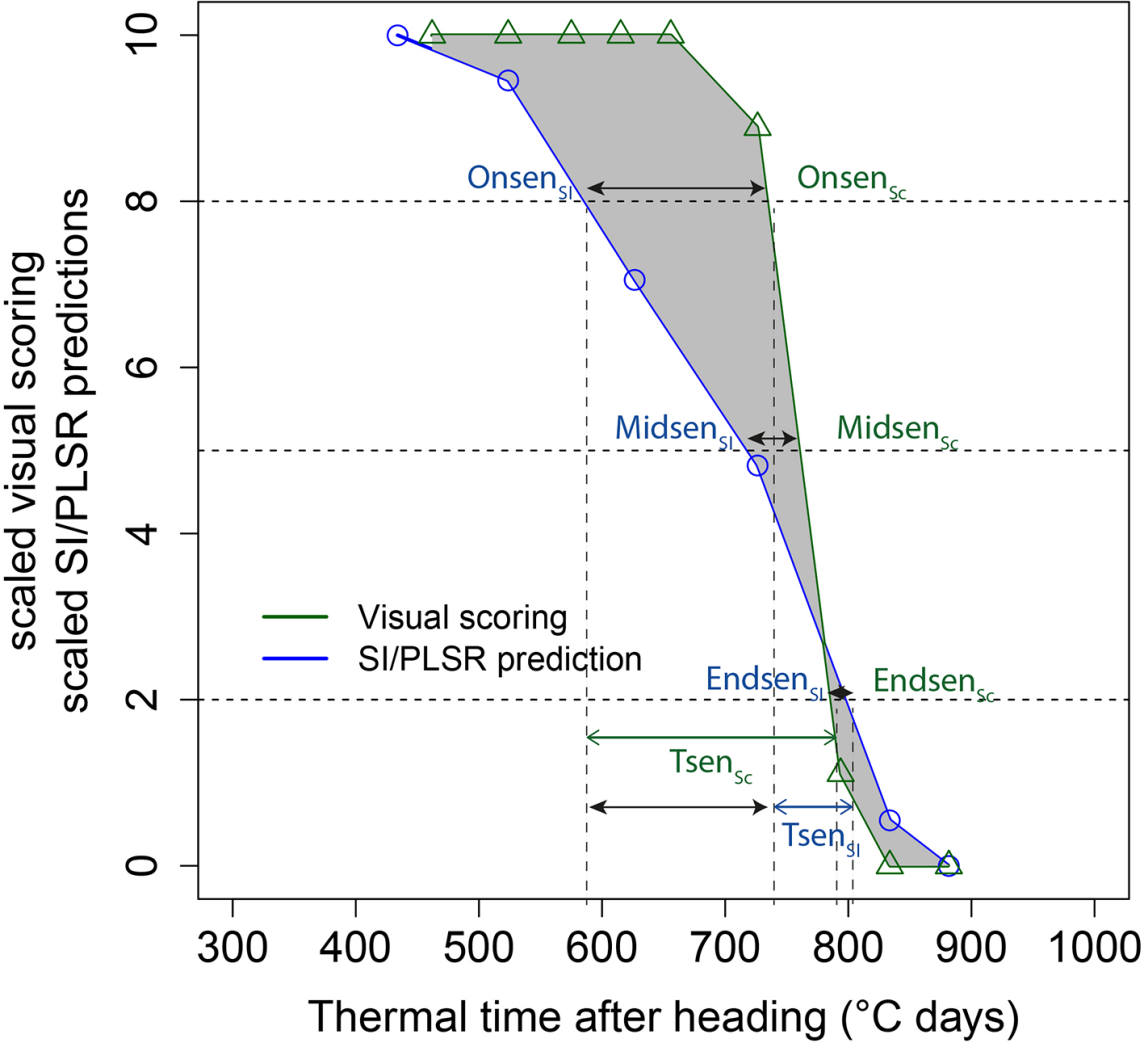
Scaled visual scorings of canopy greenness (Sc) and a scaled spectral index (SI) as a function of thermal time after heading for one experimental plot. Linear interpolation was used to derive the onset (On_sen_), mid (Mid_sen_) and end (End_sen_) of the rapid senescence phase, its duration (T_sen_) and the deviation of the SI curve from the Sc curve (error; shaded area). Black arrows represent the difference between SI- and Sc-derived parameters. The mean of these differences across all plots represents a measure of bias.

Heading date was recorded when 50% of the spikes were fully emerged from the flag leaf sheath (BBCH 59, Lancashire et al., 1991). Senescence was assessed visually, separately for the flag leaf and the whole canopy, following guidelines provided by Pask et al. (2012). Flag leaf senescence was scored based on the portion of green leaf area on a scale from 0 (0% green leaf area) to 10 (100% green leaf area). An integer mean value was estimated for plants located in a central region of about 0.5 m × 0.5 m of each plot. Whole plot senescence was scored on the same scale by estimating the overall greenness of the plot when inspected at a view angle of approximately 45° considering the entire plot area. Where necessary, the canopy was opened by hand to enable inspection of lower canopy layers. All scorings were done in 2- to 4-day intervals. Senescence scorings were done from approximately 20 days after flowering to complete canopy senescence. All heading and senescence scorings were done by the same person. The progression of leaf and whole plot senescence as assessed by visual scorings was then fitted against thermal time after heading (BBCH 59) for each individual plot using linear interpolation as well as a Gompertz model with asymptotes constrained to 0 and 10 (eq. 1; Gooding et al., 2000),

(eq. 1) $$S=10e^{-e^{-b*\left(t-M\right)}}$$

where *S* represents the scaled senescence scoring, *t* is the accumulated thermal time after heading for a given plot, *b* is the rate of senescence at time *M* and *M* is the accumulated thermal time after heading when senescence rate is at its maximum. Eq. (1) was fit for each experimental plot using the R package “nls.multstart” (Padfield and Matheson, 2018). Senescence dynamics parameters were then extracted as follows (**Figure 1**): Onset of senescence (On_sen_) was defined as the time point when values fell below 80% of the initial maximum, midpoint of senescence (Mid_sen_) when values fell below 50%, end of senescence (End_sen_) when values fell below 20%, and duration (T_sen_) was defined as the time between onset and end of senescence, similar to the procedure applied to NDVI data by Christopher et al. (2014). We will refer to the duration between heading and the onset of senescence as the duration of stay green.

GY was determined by manually harvesting the sowing rows 7 and 8 (out of 9). Grain moisture content was measured on a subset of 290 plots in 2016, 108 plots in 2017 and 84 plots in 2018, using a Wile 55 moisture meter (Farmcomp Oy; FIN-04360 Tuusula, Finland). Where available, grain weight was normalized to 14% water content using the plot-specific moisture content. The mean value of the measured plots was used otherwise. GPC was determined using near-infrared transmission spectroscopy (Infratec^TM^ 1241 Grain Analyzer; Foss, DK-3400 Hilleroed, Denmark).

### Statistical Analysis

The derived senescence dynamics parameters and agronomic traits were spatially corrected using two-dimensional P-splines as implemented in the R-package SpATS (Xose Rodriguez-Alvarez et al., 2018). To fit an independent smoothed surface to each replicate, the replicates were allocated diagonally in a grid of 49 rows by 41 ranges with replicate one ranging from row 1 to 22 and range 1 to 18 and replicate two ranging from rows 27 to 49 and range 23 to 41.The spatial model was:

(1) $$\begin{array}{c} Y_{ijkl}=f\left(r_{i},c_{j}\right)+K_{l}+G_{k}+R_{i}+\;C_{i}+\varepsilon_{ijkl} \end{array}$$

where *f* (*r_i_*, c*_j_*) is a smoothed bivariate surface defined by row *r* (*i* = 1,…,49) and range *c* (*j* = 1,…,41) as covariates (*for details see* Xose Rodriguez-Alvarez et al., 2018), *K* is the fixed effect of the check or the mean of all test genotypes (l = 1, 2, 3, $\bar{\mu }$ test), *G* is the random effect of the test genotypes (*k* = 1, …, 351), with check genotypes coded as missing. *R_i_* and *C_j_* are random factors of the rows and ranges, respectively, and ϵ is the random error vector. Twenty spline points were used each for rows and ranges.

To obtain best linear unbiased estimators (BLUEs) for all genotypes, the factor genotype was considered as a fixed effect in model (1) (*k* = 1, …, 354) and K was omitted from the model. The sum of the genotypic BLUE and the plot-specific residual error was extracted as a spatially corrected plot value.

Within-season repeatability (*w* ^2^) of the spatially corrected traits was calculated according to Xose Rodriguez-Alvarez et al. (2018) based on the genetic effective dimensions provided by SpATS as:

(2) $$\begin{array}{c} w^{2}=\frac{ED_{g}}{m_{g}-1} \end{array}$$

where ED_g_ is the effective dimension for the genotypes and m_g_ is the total number of genotypes evaluated.

Spatially corrected plot values derived from (1) were used for the multi-year model using the R package “asreml-4” (Butler et al., 2018):

(3) $$\begin{array}{c} Y_{ihkl}=\text{µ}+K_{l}+G_{i}+Y_{h}+B_{k\left(h\right)}+\;GY_{ih}+\varepsilon_{ihkl} \end{array}$$

where *Y_ihkl_* is the spatially corrected senescence dynamics parameter or single plot measurement estimated in (1), µ is the overall mean, Y the fixed effect of the year (h = 2016, …, 2018), B is fixed effect of the replication within year h (k = 1, 2), GY_ih_ the random genotype-by-year interaction and ϵ_ihkl_ is the random normally distributed error with a year-specific variance. The effect of the replicate was specified only for years where more than one replicate was measured (i.e. for reflectance-based traits, where both replicates were measured only in 2016).

Across-year heritability was derived according to the method proposed by Cullis et al. (2006) as:

$$H_{C}^{2}=1-\frac{{\text{avsed}}^{2}}{2{\hat{\sigma }}_{G}}$$

where $H_{C}^{2}$ is the heritability that is appropriate for complex residual structures (though not needed here) and avsed is the average standard error of prediction differences provided by the predict.asreml function. In the original equation provided by Cullis et al. (2006), the avsed is expressed as the mean variance of a difference between a pair of genotype ${\bar{v}\mathrm{BLUP}}_{\mathrm{diff}}$, the square of avsed (Isik et al., 2017).

### Hyperspectral Assessment of Senescence Dynamics

#### Hyperspectral Reflectance Measurements

Canopy hyperspectral reflectance in the optical domain from 350 to 2500 nm was measured using a passive spectroradiometer (ASD FieldSpec® 4 spectroradiometor; ASD Inc., USA) equipped with an optic fiber with a field of view of 25˚. Whenever possible, measurements were carried out between 10:00 and 14:00 local time under clear and cloudless conditions. However, given the need for frequent measurements and the geographic location of the experiment, this was not always possible. Reflectance spectra were recorded as the average of 15 to 25 separate spectral records. Measurements were taken from nadir view holding the sensor at a height of approximately 0.4 m above the canopy. In 2016, reflectance spectra were recorded for one to two locations per plot holding the sensor in a nadir position above a crop row. In 2017 and 2018, 5 spectra were recorded while moving the fiber optic along the diagonal of each plot. This change in the measurement procedure was decided to reduce the variance of reflectance measurements due to plot heterogeneity in senescence observed in the first year. A Spectralon® white reference panel was used for calibration before measuring canopy reflectance, and the calibration was repeated approximately every 10 min. Under more variable conditions, the device was re-calibrated more frequently. When light conditions changed perceivably, measurements were interrupted immediately, and the device was recalibrated before continuing the measurements under stable light conditions. In 2016, both replicates were measured, requiring about 3 h on average, whereas in 2017 and 2018 measurements were limited to one replicate, requiring about 2 h on average. The experiments were measured between heading and physiological maturity on 7 dates in 2016, on 8 dates in 2017 and on 12 dates in 2018. Thus, the frequency of spectral measurements was slightly lower than the frequency of visual scorings. The resulting hyperspectral dataset was then analyzed from two different perspectives relating to the main objectives of this study (**Figure 1**, upper and lower panels, respectively). The two approaches are described in more detail in the following sections and in **Supplementary Methods**. For ease of notation, reflectance at specific wavelengths will be abbreviated as R followed by the wavelength (e.g. R750).

#### Spectral Indices and Full-Spectrum Models to Infer Senescence Dynamics

**Figure 2:**
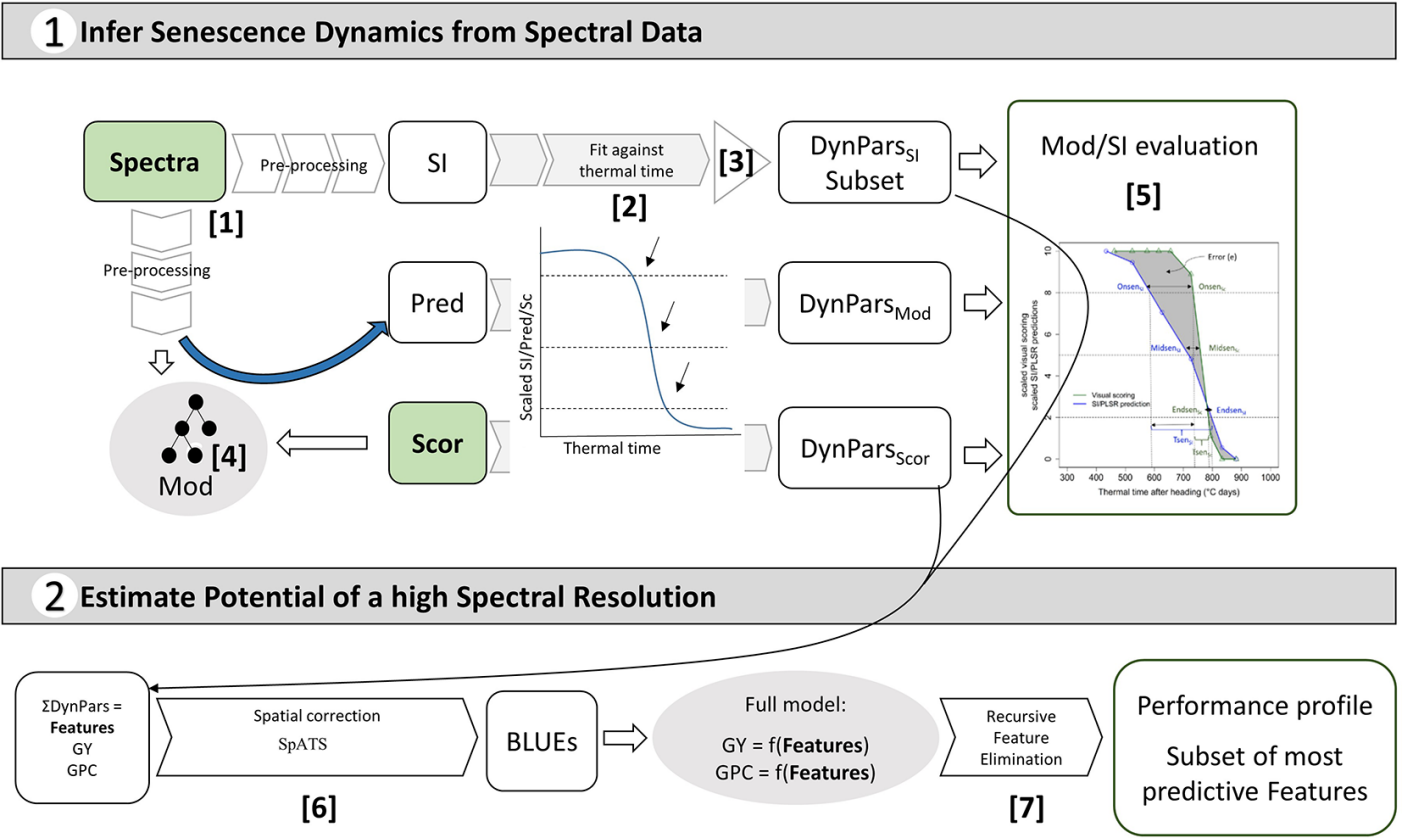
Overview of the objectives of this study and the implemented workflow: pre-processing of reflectance spectra and conversion to spectral indices (SI) [1]; full-spectrum models (Mod) to obtain predictions (Pred) of visual senescence scorings (Sc) based on reflectance spectra [4]; fitting of SI, Sc and Pred against thermal time and extraction of corresponding dynamics parameters (DynPars) [2]; Unsupervised DynPars_SI_ subset selection [3]; Model and SI evaluation based on DynPars [5]; Spatial correction and calculation of best linear unbiased estimators (BLUEs) [6]; Modelling of primary traits (i.e. grain yield (GY) and grain protein concentration (GPC)) and supervised feature selection by recursive feature elimination [7] to determine the most predictive features and estimate the potential benefits of a high spectral resolution.

**Figure 3:**
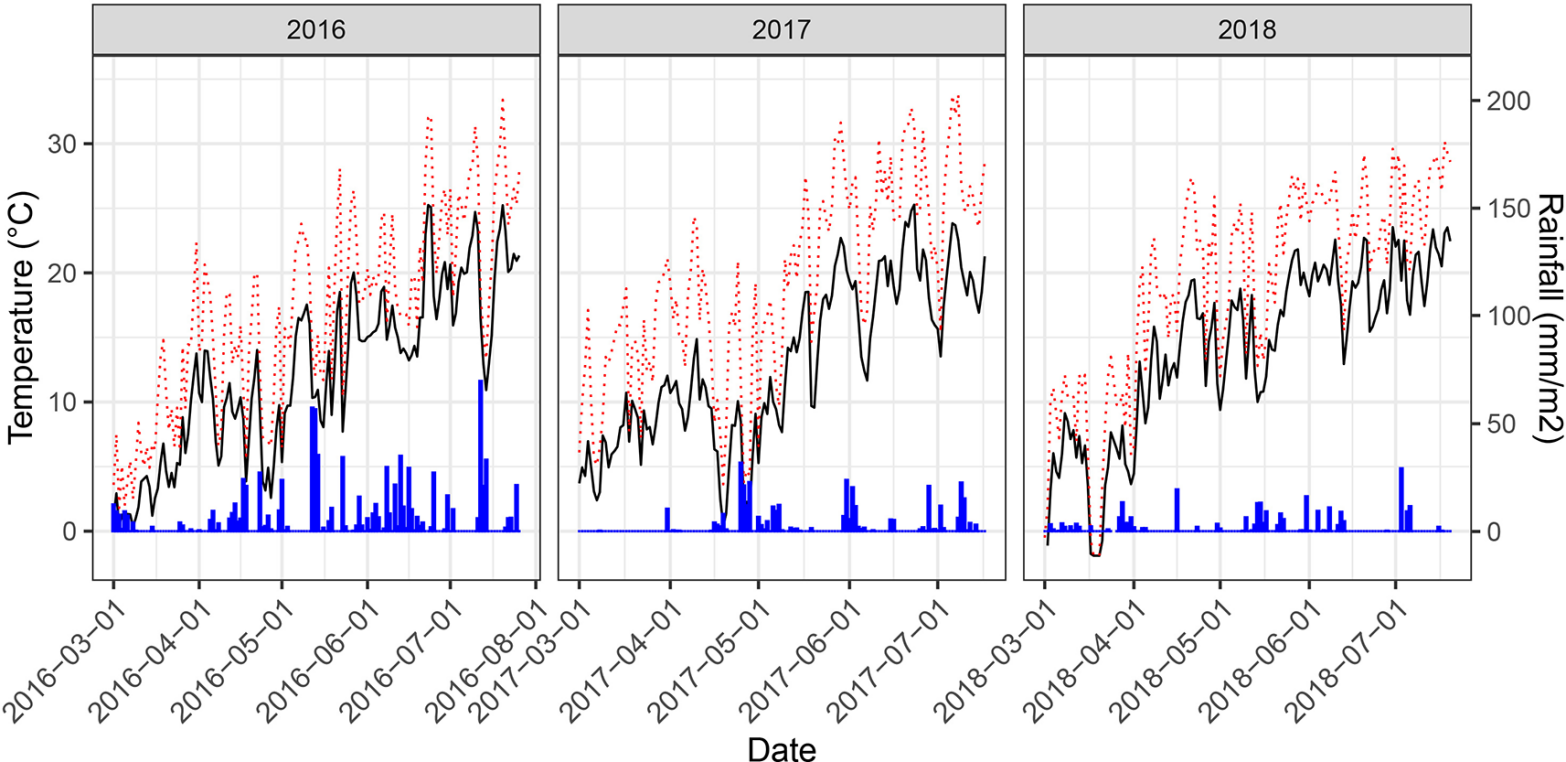
Daily mean temperatures (black solid line), daily maximum temperatures (red dotted line) and rainfall measured at 2 m above the ground for the main growing period of the experiment at the field phenotyping platform of ETH Zurich. Temperature data was retrieved from an on-site weather station. Rainfall data was obtained from a nearby weather station of the federal Swiss meteorological network Agrometeo (www.agrometeo.ch).

An assessment of the performance and robustness of SI and full-spectrum models to track canopy senescence across environments was performed. A detailed description of the methodology is provided in **Supplementary Methods**. In brief, a large number of published spectral indices were computed (**Table S1**) and full-spectrum models to infer visually observed senescence scorings were calibrated from pre-processed reflectance spectra (**Figure 2**, [1]). Models were used to generate predictions of senescence scorings for unseen data of the same environment as used in model calibration and of environments not included in model calibration. The resulting SI values and model predictions were scaled to range from 0 to 10, representing the minimum and maximum value recorded or predicted for the assessment period, respectively. Scaled values were fitted against thermal time after heading, and parameters describing the observed dynamics were extracted from time courses as was done for visual scorings (**Figure 2**, [2]). A subset of spectral indices was then selected using several filtering criteria to reduce multi-collinearity of the dataset (**Figure 2**, [3]). For full-spectrum models, waveband selection was performed in each experiment using recursive feature elimination. Performance and robustness of selected SI and full-spectrum models was assessed by comparing the dynamics parameters obtained from selected SI and model predictions to those obtained from visual scorings as shown in **Figure 1** (step [5] in **Figure 2**). Pearson product moment correlation coefficients were calculated for the dynamics parameters obtained from linear interpolation of visual scorings, SI and model predictions. The mean difference in GDD over all experimental plots between the SI-derived and the scoring-derived parameters was also calculated to reveal potential general bias. Finally, the area between the resulting lines was calculated as a measure of precision in tracking the entire process.

#### Multiple Spectral Indices During Senescence to Predict Primary Traits

Finally, all senescence dynamics parameters obtained from scorings and from the selected SI (hereafter referred to as features) were analyzed directly for their association with GY and GPC. BLUEs or spatially corrected values were used for the analysis (**Figure 2**, [6]). We aimed to answer three separate questions in a step-wise procedure: First, whether a phenotypic correlation between senescence dynamics and GY and GPC existed for any given trait in any given year; we used simple linear regression models for this purpose. Second, if the results suggested the presence of such a linear correlation, we investigated the potential of additional information contained in multiple SI time courses as opposed to the time course of a single SI or visual scoring. Such single SI or scoring values are likely to capture only part of the changes occurring during senescence (e.g. the dynamics of chlorophyll breakdown) while other processes might hold complementary information. Third, we aimed at identifying the most important features to predict the trait. The rationale behind this was the following: Given a significant correlation between senescence dynamics and GY and GPC and a number of features describing aspects of senescence, the feature identified by the model to be the most relevant feature to predict GY or GPC should also be the one feature that most precisely captures the relevant aspects. For this purpose, we conducted supervised feature selection by recursive feature elimination (**Figure 2**, [7]; *see* Ambroise and McLachlan, 2002; Guyon et al., 2002; Granitto et al., 2006 *for a detailed description and discussion of the methodology*). A detailed description is provided in **Supplementary Methods**.

## Results

### Experiments Represented Contrasting Environments

Weather conditions during the main growing phase of the three experimental years strongly contrasted (**Figure 3**). The year 2016 was characterized by a wet summer with high precipitation causing severe lodging and high levels of foliar diseases. In particular, high levels of Septoria tritici blotch (STB) caused by the fungal pathogen *Zymoseptoria tritici* were observed. A total of 88 plots had to be excluded from further analyses due to heavy lodging. An additional 24 plots were excluded due to extended patches affected by take-all disease (*Gaeumannomyces graminis* var. *tritici*), which made objective senescence scorings and reflectance measurements impossible. Contrarily, the years 2017 and 2018 were characterized by dry summers and in 2017 additionally by high temperatures with daily maximum temperatures exceeding 30°C on several days, particularly during grain-filling. While both biotic and abiotic stresses can affect senescence dynamics in wheat, the underlying responses are stress-specific and may be controlled by very different genes or gene networks (Guo and Gan, 2012). Consequently, the three experimental years can be considered contrasting environments for the assessment of senescence dynamics and effects on GY and GPC.

### Large Variability and Moderate to High Heritability for Senescence Dynamics and Agronomic Traits

Large variability was observed for heading date, GY and GPC among the >330 genotypes in all years (**Table 1**). Heading occurred 8 days earlier in 2018, likely due to the comparably dry conditions in spring (**Figure 3**). Large variability was also observed for senescence dynamics, with a difference of >300 °C days in the onset between the earliest and the latest genotype in all years. Similarly, the duration of senescence varied strongly across genotypes. The rate of senescence was somewhat lower in 2017 as expressed by an increased duration of the process. Across all genotypes, flag leaf senescence was somewhat delayed with respect to canopy senescence in 2016 and 2017, especially in early senescing genotypes. In 2018, this sequential vertical pattern of senescence was much less pronounced (data not shown). The stay-green phase was shorter in the dry seasons of 2017 and 2018 and longer in the wet season of 2016. Correlations between the senescence dynamics parameters extracted from the non-linear model fit and linear interpolation of visual senescence scorings were high for On_sen_ (r = 0.94), Mid_sen_ (r = 0.99) and End_sen_ (r = 0.96), suggesting a good approximation of the dynamic patterns through linear interpolation. Therefore, linear interpolation was used for further analyses. Repeatability for senescence dynamics parameters was higher in 2016 than in 2017 and intermediate in 2018 (**Table 1**). Repeatability for On_sen_, Mid_sen_, and End_sen_ was moderate to high, ranging from 0.72 to 0.79, from 0.44 to 0.65 and from 0.64 to 0.68 in 2016, 2017 and 2018, respectively. For T_sen_, repeatability was distinctly lower in all years. Across-year heritabilities were intermediate to high for On_sen_, Mid_sen_, and End_sen_(**Table 1**).

**Table 1:** Descriptive statistics, within-year repeatability and across-year heritability for heading date (HD), grain yield (GY), grain protein concentration (GPC) and senescence dynamics parameters derived from linearly interpolated visual canopy senescence scorings (Sc,Lin) and from linearly interpolated values of the PSRI (PSRI,Lin).

	Mean (Min; Max)	w^2^	h^2^ (2016–2017–2018)
**HD (Julian Day)**	152 (146; 158)/ 152 (145; 157)/ 144 (137; 150)	0.96/ –/ –	0.97
**GY (t ha** ^−^ **^1^)**	4.66 (2.81; 6.86)/ 5.39 (3.40; 7.55)/ 5.63 (2.92; 8.16)	0.54/ 0.40/ 0.41	0.55
**GPC (%)**	12.7 (10.2; 15.8)/ 12.9 (10.7; 15.8)/ –	0.83/ 0.70/ –	0.84*
**Onsen_Sc,Lin_**, **°C (days)**	647 (435; 854)/ 603 (353; 748)/ 598 (447; 748)	0.72/ 0.44/ 0.64	0.60
**Midsen_Sc,Lin_**, **°C (days)**	701 (544; 894)/ 675 (456; 815)/ 646 (487; 789)	0.78/ 0.65/ 0.68	0.75
**Endsen_Sc,Lin_**, **°C (days)**	748 (592; 926)/ 730 (516; 854)/ 706 (529; 873)	0.79/ 0.62/ 0.64	0.76
**Tsen_Sc,Lin_**, **°C (days)**	101 (28; 258)/ 128 (49; 322)/ 108 (26; 301)	0.22/ 0.24/ 0.24	−0.80
**Onsen_PSRI,Lin_**, **°C (days)**	–	0.75/ –/ –	0.57
**Midsen_PSRI,Lin_**, **°C (days)**	–	0.79/ –/ –	0.77
**Endsen_PSRI,Lin_**, **°C (days)**	–	0.84/ –/ –	0.76
**Tsen_PSRI,Lin_**, **°C (days)**	–	0.74/ –/ –	0.06

*Heritability for GPC is based on data from 2016 and 2017 only. Data referring to individual years is reported sequentially for consecutive years (2016/2017/2018).

### Spectral Reflectance Is Associated With Visual Senescence Scorings in a Non-Linear Manner

**Figure 4:**
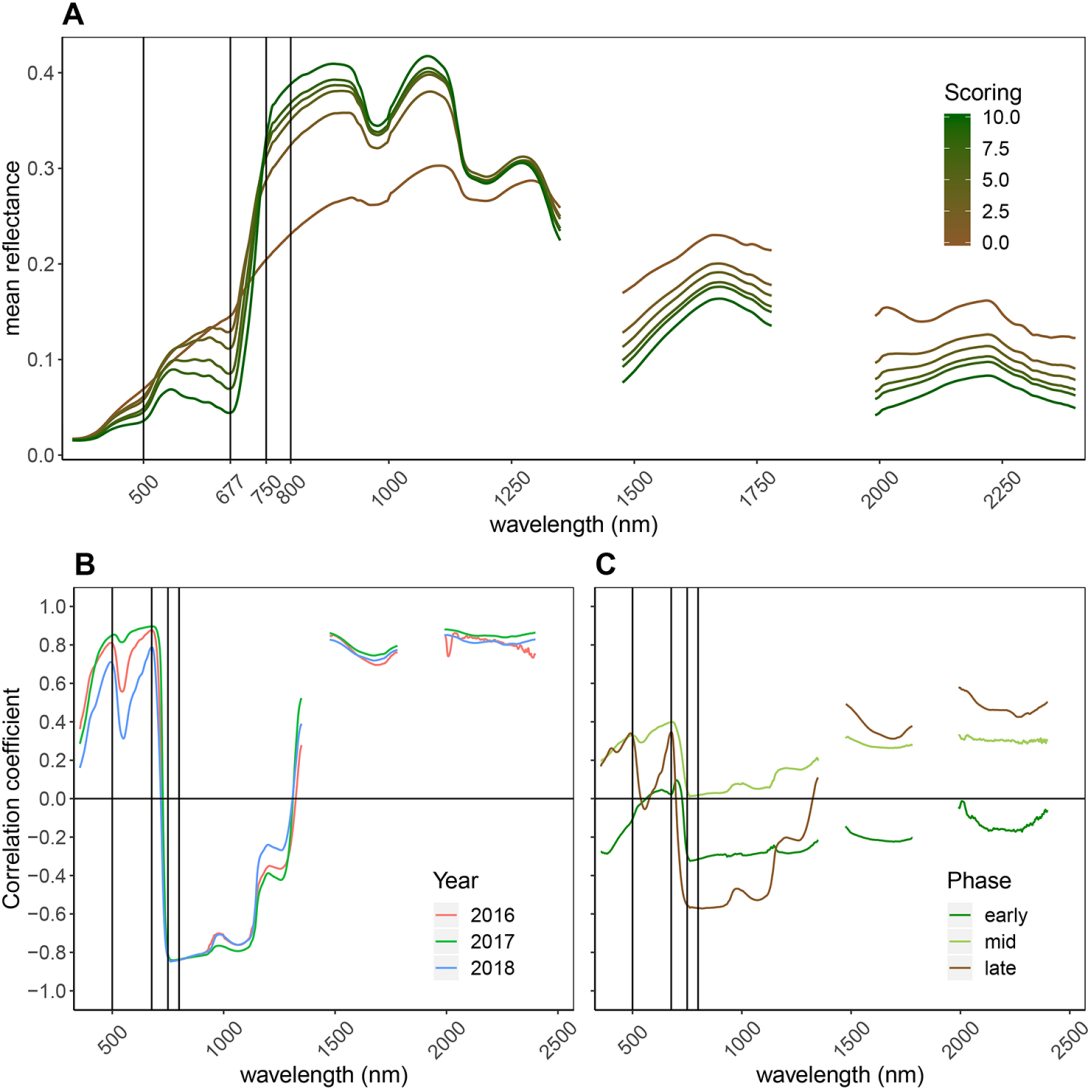
General reflectance patterns of senescing wheat canopies. **(A)** Mean reflectance spectrum of wheat genotypes through the process of senescence (10 denotes completely green canopies, 0 denotes complete senescence, based on visual scorings). Data from all time points and all years was used to calculate the mean reflectance spectrum per scoring. Vertical lines mark the wavebands constituting the NDVI and the PSRI. **(B)** Pearson correlation between reflectance at each wavelength and visual senescence scorings. Positive correlations indicate increasing reflectance as senescence progresses, negative correlations indicate decreasing reflectance as senescence progresses; Year-specific correlation coefficients; **(C)** Separate analyses for early senescence (scorings = [0:3]), intermediate senescence (scorings = [4:7] and late senescence (scorings = [8:10]). This part of the graph is based on data of the 2018 experiment.

Senescence led to major changes in canopy reflectance throughout the recorded spectrum (**Figure 4A**). Reflectance in the visible range (VIS; 400–700 nm) increased strongly, whereas reflectance in the near infrared (NIR; 750–1300 nm) portion of the spectrum decreased. In the short-wave infrared (SWIR; 1,475–1781 nm and 1991–2400 nm) portion of the spectrum, reflectance increased. Pearson correlation coefficients between the reflectance at each wavelength and the visual canopy senescence scores were calculated for each year (**Figure 4B**) and separately for different phases of the senescence process (**Figure 4C**). High positive correlations were found between the reflection in the VIS, with peaks at around 500 and 680 nm, as well as in the SWIR, indicating a decrease of light absorption (resulting in an increase in reflection) in these parts of the spectrum as senescence progresses. Strong negative correlations were found in the NIR with a peak near 750 nm, indicating a strong decrease of reflectance in this portion of the spectrum as senescence progresses. These patterns were consistent across years. When different phases of the senescence process were analyzed separately, major differences in the correlations over large parts of the spectrum were found, indicating that reflectance throughout the spectrum is associated in a non-linear manner with visual senescence scorings.

### Spectral Indices Track Visually Observed Senescence Dynamics Across All Years

**Figure 5:**
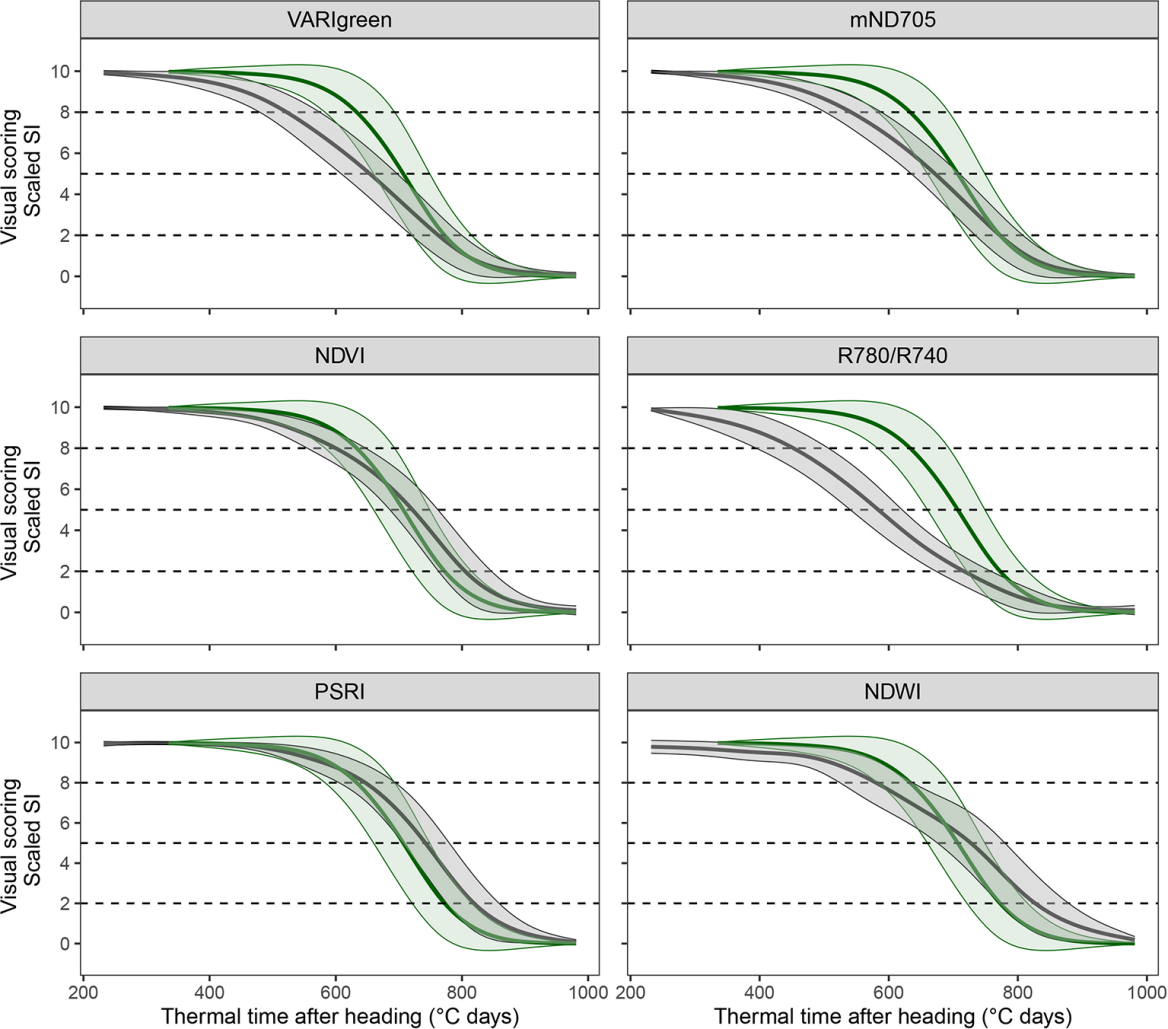
Dynamic pattern of scaled spectral indices (in grey) and visual canopy senescence scores (in green; identical in all subplots) over thermal time after heading. Mean linearly interpolated values over all experimental plots of the 2016 experiment (thick lines) and their standard deviations (thin lines) are shown. Dashed lines mark the thresholds defined as onset, midpoint and end of senescence.

**Figure 6:**
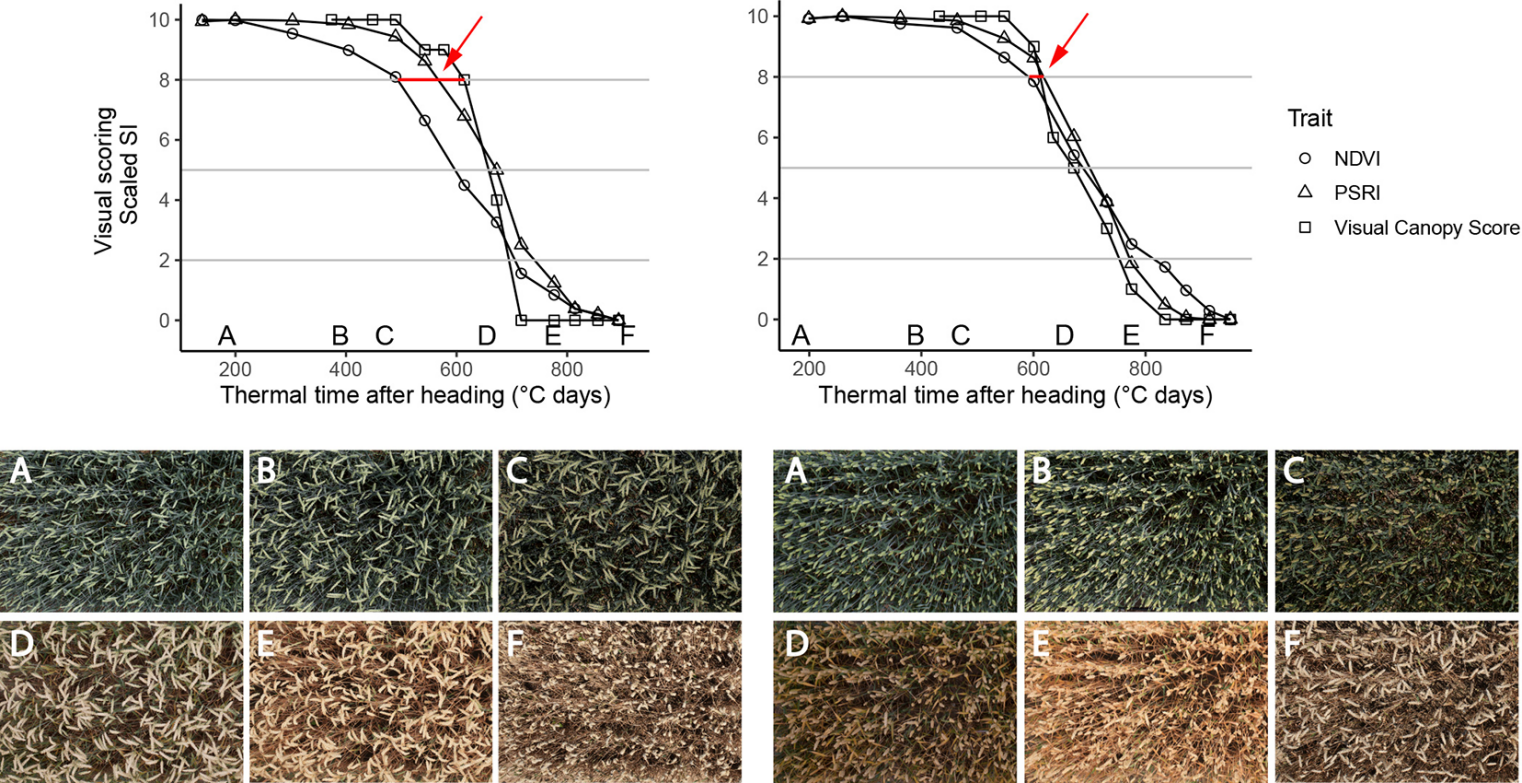
Time courses of PSRI, NDVI (nadir view) and visual scorings (whole plot, 45° viewing angle) for two experimental plots of the 2018 experiment. Left: Genotype with changing spike orientation during grain filling. With time, spikes make up an increasingly dominant part of the image. Concomitantly, NDVI values decrease early in the grain filling phase, while visual scorings indicate no change in canopy greenness (evidenced by red arrow). Right: Genotype characterized by relatively stable spike orientation during grain filling and comparable NDVI, PSRI and scoring time courses (evidenced by red arrow); Letters A-F in the upper part of the Figure represent time points when corresponding images were taken. Images were taken by the field phenotyping platform (FIP, Kirchgessner et al., 2017).

A subset of 83 SI-derived senescence dynamics parameters was retained for further analyses. These included 21 T_sen_ parameters, suggesting that this parameter could be measured with a satisfactory repeatability (w^2^ > 0.5) using certain SI. Several SI could be identified for which the mean value across all experimental plots followed clearly contrasting dynamic patterns (*see* **Figure 5** *for examples*). Generally, the NDVI-derived senescence dynamics parameters correlated well with the scoring-derived parameters. However, for some SI the senescence parameters consistently correlated better with the scoring-derived parameters and were less biased (i.e. deviated less from scorings) than the NDVI-derived parameters (**Table 2**, **Figure 5**). PSRI-derived onset of senescence correlated best with scoring-derived onset and was unbiased (r = 0.72, d_Onsen_ = 6°C days, r = 0.78 and d_Onsen_ = −11°C days and r = 0.75, d_Onsen_ = −7°C days for 2016–2018, respectively) as opposed to the parameter derived from NDVI (r = 0.64, d_Onsen_ = −43°C days, r = 0.63 and d_Onsen_ = −61°C days and r = 0.51, d_Onsen_ = −57°C days in 2016–2018, respectively). PSRI also predicted midpoint of senescence with a high accuracy (r = 0.76, d_Midsen_ = 43°C days, r = 0.91, d_Midsen_ = 25°C days and r = 0.86, d_Midsen_ = 26°C days in 2016–2018, respectively). Endpoint of senescence was predicted quite accurately (r ≈ 0.7 across all years) by several SI, whereas the NDVI was clearly less stable across different years. Across all three years, End_sen_ derived from VARIgreen was correlated best with scoring-derived End_sen_ (r = 0.79, d_Endsen_ = 10°C days, r = 0.83, d_Endsen_ = −18°C days and r = 0.82, d_Endsen_ = −17°C days in 2016–2018, respectively), while NDVI was clearly less precise and less stable across years (r = 0.59, d_Endsen_ = 53°C days, r = 0.78, d_Endsen_ = 65°C days, and r = 0.54, d_Endsen_ = 52°C days in 2016–2018, respectively). The PSRI performed best in tracking the senescence process as observed visually from the onset to the end of the process, as expressed by comparably small error reprinted by the area between the curves (**Table 2**). Since repeatability for T_sen_ assessed visually was low in all years, results of the correlation analysis should be interpreted with caution, but the strongest correlations were found again for the PSRI. Thus, in summary, the PSRI outperformed the NDVI and all other tested SI in assessing most of the senescence dynamics parameters investigated here. Importantly, the observed correlations were stable across the three years. A comparison of experimental plots for which NDVI-derived On_sen_ strongly differed from the scoring-derived On_sen_ with RGB images suggested that this might be largely due to canopy structural effects such as leaf angles, spike geometry and spike orientation. This is illustrated in **Figure 6** for two contrasting example plots, sown respectively with a genotype with changing spike orientation during grain filling (**Figure 6**, right) and a genotype with relatively stable spike orientation (**Figure 6**, left).

**Table 2:** Pearson correlation (*p < 0.05; **p < 0.01; ***p < 0.001) between the senescence dynamics parameters derived from visual scorings and spectral indices, mean deviation in GDD between the derived parameters, and the total error throughout the entire process.

Index	r_Onsen_	Bias_Onsen_ (°Cd)	r_midsen_	Bias_Mnsen_ (°Cd)	r_Endsen_	Bias_Endsen_ (°Cd)	r_tsen_	Bias_tsen_ (°Cd)	Mean error
**mND705**	0.50***/0.61***/0.44***	−101/−128/−122	0.81***/0.83***/0.67***	−28/−45/−50	0.77***/0.84***/0.69***	22/5/−11	0.10*/0.04/0.10*	124/132/111	569/703/664
**PRInorm**	0.64***/0.76***/0.63***	41/23/19	0.53***/0.83***/0.67***	71/68/79	0.47***/0.56***/0.30***	82/119/104	0.16***/0.47***/0.03	41/96/85	686/787/767
**PSND1**	0.64***/0.63***/0.51***	−41/−59/−55	0.76***/0.84***/0.66***	25/8/5	0.59***/0.78***/0.55***	54/65/53	0.22***/0.17**/0.04	95/125/107	497/578/539
**PSRI**	0.72***/0.78***/0.75***	6/−11/−7	0.76***/0.91***/0.86***	43/25/26	0.63***/0.85***/0.81***	64/62/43	0.27***/0.25***/0.07	58/73/49	498/473/393
**VARIgreen**	0.44***/0.49***/0.25***	−121/−175/−135	0.71***/0.71***/0.56***	−45/−72/−49	0.79***/0.83***/0.82***	10/−18/−17	0.05/−0.05/0.03	131/157/117	648/898/716
**NDVI**	0.64***/0.63***/0.51***	−43/−61/−57	0.76***/0.83***/0.66***	24/7/3	0.59***/0.78***/0.54***	53/65/52	0.22***/0.16**/0.05	96/126/109	498/581/543

Only spectral indices outperforming the NDVI in all three years for at least one parameter are listed, and the respective cells are highlighted in green. Several additional Spectral indices gave a better representation of End_sen_, but only the VARIgreen, which performed best, is listed here.

### Full-Spectrum Models Are Environment-Specific

We aimed to develop a further optimized spectral model to track wheat canopy senescence exploiting the full spectrum. Both tested algorithms resulted in significantly improved predictions of senescence scorings compared to the best SI for held out samples of the same year. This resulted in smaller errors in tracking the entire process (**Tables 2** and **3**).

**Table 3:** Within-year and across-year validation results for partial least squares regression (PLSR) and cubist regression models.

Algorithm	Type of validation	Train Experiment	Validation Experiment	RMSE	r_onsen_	r_midsen_	r_endsen_	r_tsen_	e
PLSR	within year	2016	2016	1.01 (1.15)	0.50	0.81	0.71	0.12	412
PLSR	within year	2017	2017	0.82 (1.00)	0.75	0.88	0.76	0.09	425
PLSR	within year	2018	2018	0.0.81 (1.06)	0.81	0.88	0.83	0.22	231
PLSR	across year	2017	2016	3.81 (3.65)	0.21	0.30	0.25	0.05	712
PLSR	across year	2018	2016	3.19 (3.12)	0.21	0.43	0.59	−0.03	518
PLSR	across year	2017, 2018	2016	4.77 (4.58)	0.15	0.30	0.37	−0.09	658
PLSR	across year	2016	2017	2.42 (3.23)	0.63	0.85	0.81	−0.05	522
PLSR	across year	2018	2017	2.29 (2.21)	0.53	0.85	0.81	−0.27	511
PLSR	across year	2016, 2018	2017	1.76 (1.92)	0.53	0.84	0.83	−0.10	559
PLSR	across year	2016	2018	2.08 (2.42)	0.79	0.87	0.83	0.14	361
PLSR	across year	2017	2018	1.36 (1.57)	0.76	0.81	0.73	0.10	412
PLSR	across year	2016, 2017	2018	1.42 (1.66)	0.79	0.85	0.83	0.19	363
cubist	within year	2016	2016	0.78 (0.87)	0.76	0.85	0.85	0.30	290
cubist	within year	2017	2017	0.66 (0.82)	0.81	0.89	0.79	0.06	371
cubist	within year	2018	2018	0.66 (0.74)	0.83	0.89	0.86	0.29	171
cubist	across year	2017	2016	0.98 (1.44)	0.30	0.53	0.52	−0.02	464
cubist	across year	2018	2016	1.80 (2.31)	0.29	0.56	0.66	−0.01	456
cubist	across year	2017, 2018	2016	1.80 (2.24)	0.31	0.46	0.54	0.04	458
cubist	across year	2016	2017	1.03 (1.25)	0.76	0.85	0.80	−0.02	441
cubist	across year	2018	2017	1.46 (1.94)	0.76	0.86	0.79	−0.06	454
cubist	across year	2016, 2018	2017	1.25 (1.74)	0.77	0.86	0.81	−0.10	455
cubist	across year	2016	2018	1.17 (1.54)	0.82	0.86	0.87	0.25	200
cubist	across year	2017	2018	0.98 (1.44)	0.82	0.85	0.84	0.15	243
cubist	across year	2016, 2017	2018	0.97 (1.42)	0.82	0.86	0.86	0.23	224

Results are shown for smoothed reflectance spectra as input data. Data was mean-centered and scaled to unit variance prior to modeling. Root mean square error (RMSE) of the scoring predictions, Pearson correlation coefficients between the senescence dynamics parameters derived from visual scorings and full-spectrum models, and the total error in assessing the entire process (area between the curves) are shown. Cases where the full-spectrum models outperformed the PSRI are highlighted in green, other cases are highlighted in red. As the main interest lies on the capability of full-spectrum models to represent the entire process of senescence, an average RMSE for 10 different random upsamples of the test data are reported in brackets, where each upsample contains all possible scoring values an equal number of times, i.e., exactly the number of times of the most frequent observations.

**Figure 7:**
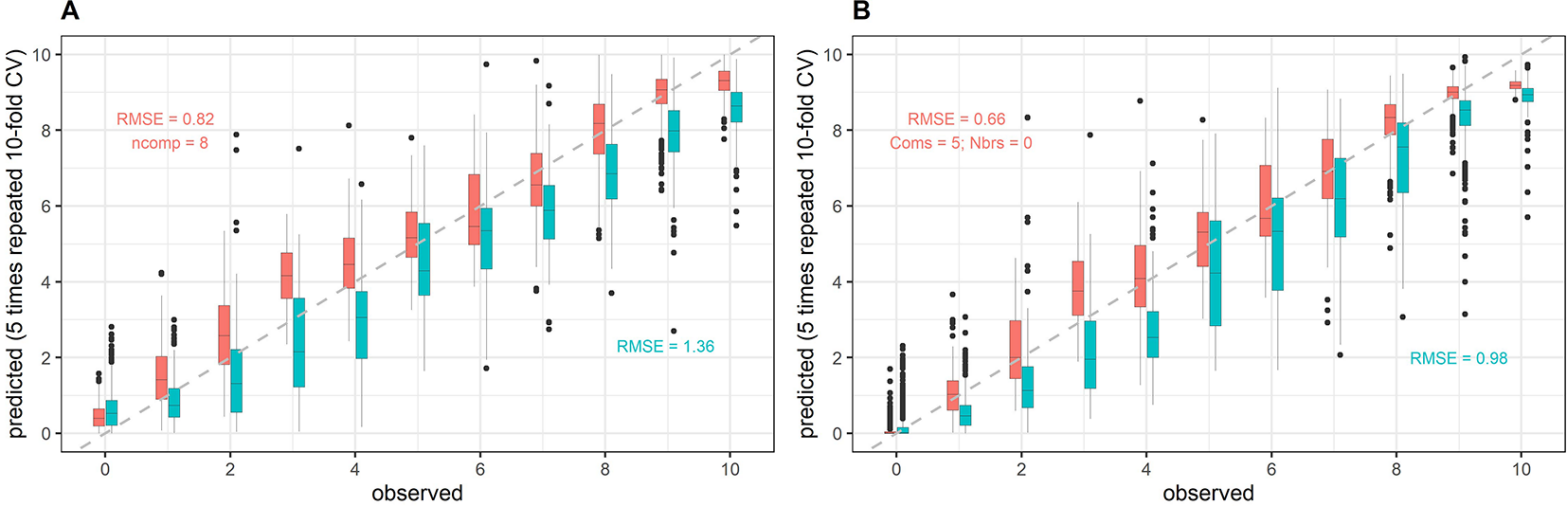
Example of model within-year and across-year validation results. Predictions of senescence scorings obtained from full-spectrum models are plotted against the visual scorings (observed). Here, averaged reflectance spectra were used, and the data was mean-centered and scaled to unit variance prior to modeling. Data from the 2017 experiment was used for model training. Models were validated on held-out samples of the same year (within-year validation, red) as well as on samples from the 2018 experiment (across-year validation, cyan). The full dataset was used for model training, i.e. no down-sampling was performed, whereas validation datasets were randomly down-sampled. **(A)** Results for partial least squares regression; **(B)** Results for cubist regression.

Cubist regression models performed better and reduced the RMSE by an average of 0.2 with respect to the PLSR models (**Table 3**). Overall, PLSR-derived senescence dynamics parameters were not higher correlated with scoring-derived parameters than the SI-derived parameters (**Table 3**). In contrast, cubist produced better estimates of On_sen_, Mid_sen_, and End_sen_ and outperformed the SI in most cases. The difference between algorithms was particularly ample in 2016. However, when models were validated across years, correlations were drastically reduced in many cases. Major differences were found for accuracy in predicting all senescence dynamics parameters, depending on which year(s) were used for training and validation, respectively. Generally, adding a second year to the training data did not substantially improve model performance on samples of the held out year. In some cases, the correlations were even negatively affected by adding additional training data, especially when PLSR was used. Commonly observed problems were (i) remaining non-linearity in the predicted vs. observed regressions, particularly for PLSR (**Figure 7A**), and (ii) year-specific bias in the predicted vs. observed regression (**Figures 7A, B**). Neither data type or pre-processing procedure was clearly and consistently superior to another. Notably, the year-specific bias could not be removed by using first derivatives or continuum-removed spectra.

**Figure 8:**
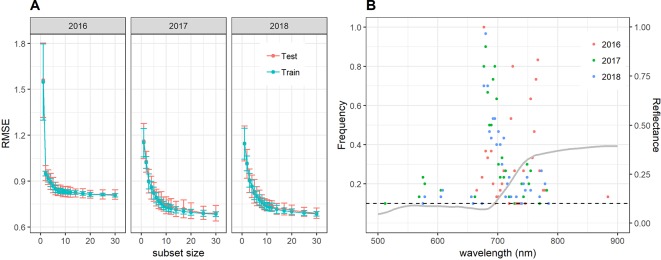
**(A)** Performance of the cubist regression models to predict visual senescence scorings depending on the number of wavelengths used as predictors. Mean performance as measured by the RMSE of predictions and standard deviations are shown based on 30 resamples of the data. **(B)** Frequency of wavelengths resulting among the most informative to predict visual scorings of canopy senescence. Frequencies denote the number of times out of 30 resampling iterations in which a given wavelength was retained in the cubist regression model down to a subset size of 12 wavelengths during recursive feature elimination. Only wavelengths which were among the top 12 predictors in at least 10% of the resamples (i.e. in at least 3 resamples, marked by the dashed horizontal line) are shown. The grey line represents the mean reflectance spectrum of canopies with a visual scoring of 8 (early senescence).

**Figure 9:**
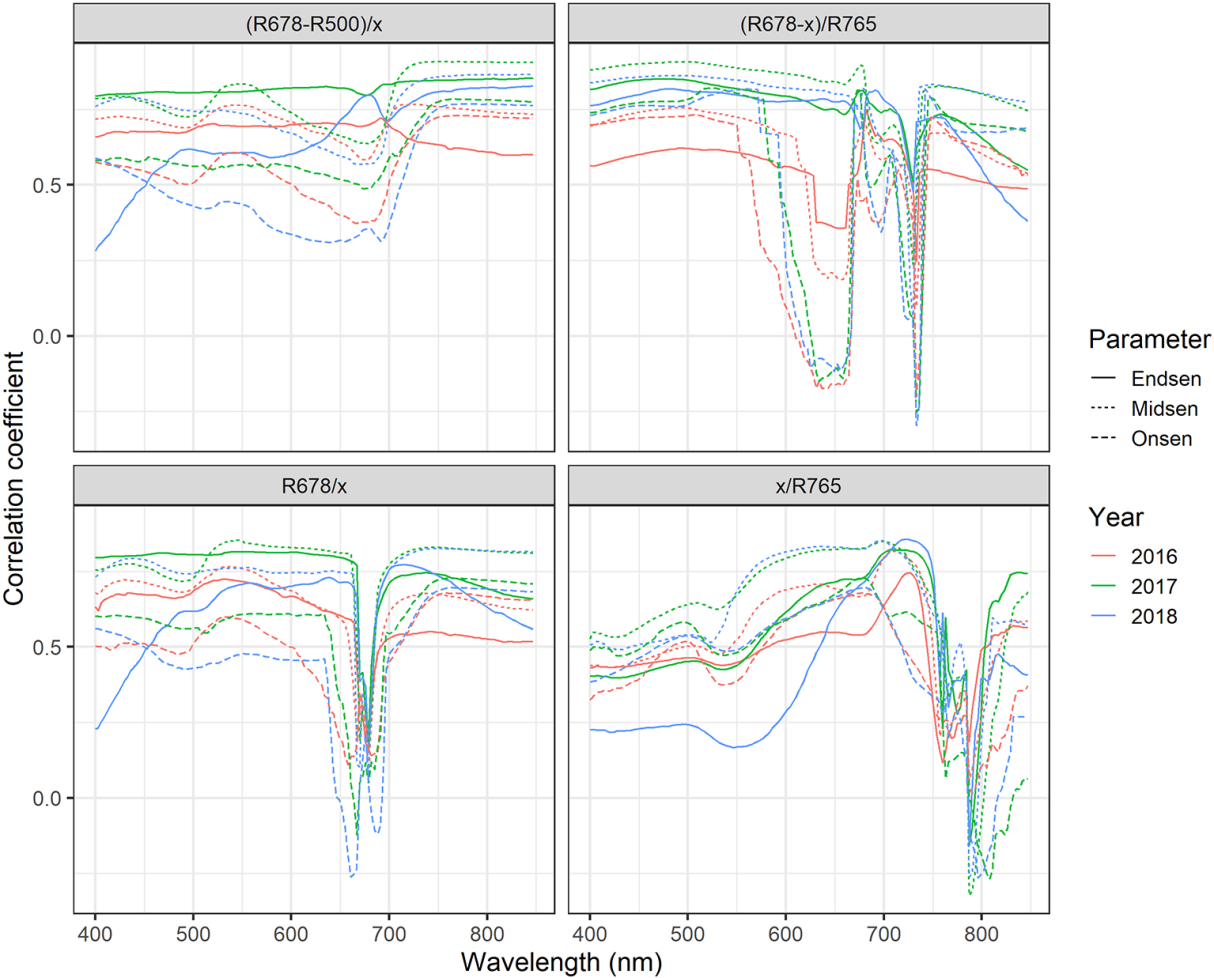
Correlation-based sensitivity analysis of the spectral bands (500, 678 and 756) constituting the PSRI. The “x” in the SI formula denotes the reflectance at the waveband that was varied in the depicted range.

Near-optimal models could be created using six to eight wavelengths for 2016 and 12 to 14 wavelengths for 2017 and 2018 (**Figure 8A**). In all years, most of the commonly selected wavelengths were contained in the 650 nm to 800 nm range (**Figure 8B**). However, there were some obvious differences between 2016, on the one hand, and 2017 and 2018, on the other hand. Models for 2016 frequently used several wavelengths between 720 and 770 nm, whereas models for 2017 and 2018 relied more heavily on the region from 670 to 720 nm, i.e., the chlorophyll absorption maximum and the red edge. Models for 2016 used a combination of R677 and one wavelength in the NIR (most often 764 nm or 767 nm) as the top two predictors in all 30 resamples, and this combination contained most of the spectral information (**Figure 8A**). Contrarily, models for 2017 and 2018 used several wavelengths (typically 3–6) in the range from 677 nm to 695 nm before including a wavelength in the NIR or around 575 nm. Given the limited potential of full-spectrum models to infer senescence dynamics across years, we aimed to optimize the PSRI to the case of wheat canopy senescence and identify the factors driving its temporal dynamics. For this purpose, we simplified the PSRI to a simple ratio index and searched the spectrum for optimal waveband compositions for these simple ratio indices as well as for the original 3-band PSRI formula (**Figure 9**). The 750 nm waveband in the denominator of the PSRI is at the upper limit of the red edge. Moving R750 towards R800 did not significantly affect the accuracy of the index, whereas moving it into the red edge affected it negatively (**Figure 9**, upper left panel). Thus, similarly to the NDVI, the PSRI appears to be driven largely by chlorophyll absorption and canopy structure. However, omitting R500 from the PSRI (i.e. reducing the PSRI to a simple ratio index R678/R750) resulted in a decrease of its accuracy (**Figure 9**, lower left panel). Substituting R500 by neighboring wavelengths had little effect, although a small improvement was observed when replacing R500 by R525 (**Figure 9**, upper right panel).

### Grain Yield and Grain Protein Concentration Correlate With Senescence Dynamics

Simple linear regression models suggested the presence of significant, albeit rather weak, linear phenotypic correlations between senescence dynamics and GY and GPC in all years (**Table 4**). The strongest linear correlation was found between the PSRI-derived onset of senescence and GY in 2016 (r = 0.369, p < 0.001) which was slightly higher than the linear correlation between scoring-derived midpoint of senescence and GY (r = 0.365, p < 0.001) and significantly higher than the correlation between NDVI-derived onset of senescence and GY (r = 0.311, p < 0.001). A significant linear correlation was also found between T_sen_ derived from several SI and GPC in 2016 (r = −0.297, p < 0.001 for NPCI). In 2017 and 2018, there was only a weak (r < 0.19) linear correlation between senescence dynamics parameters and GY and GPC. In these years, scoring-derived parameters were always among the three most highly linearly correlated senescence dynamics parameters for both traits.

**Table 4:** Correlation (**, p < 0.01; ***, p < 0.001) between senescence dynamics parameters and grain yield (GY) and grain protein concentration (GPC) in different years.

Year	Trait	Senescence dynamics parameter	Pearson r
2016	GY	onsen_PSRI	0.369***
		midsen_NDWI1	0.365***
		midsen_gom_SnsCnp	0.365***
	GPC	tsen_NPCI	−0.297***
		tsen_HI	−0.282***
		tsen_R780/R700	−0.259***
2017	GY	onsen_NDRE	0.187***
		onsen_gom_SnsCnp	0.182***
		onsen_MSR_rev	0.172**
	GPC	midsen_SnsCnp	−0.283***
		midsen_gom_SnsCnp	−0.269***
		onsen_gom_SnsCnp	−0.245***
2018	GY	tsen_RGR	0.191***
		midsen_SnsCnp	0.177***
		onsen_SnsCnp	0.173**

Pearson correlation coefficients are reported for the three most highly correlated features for each trait × year combination.

Heading date correlated negatively with the duration of the stay-green phase. The strongest correlation was observed in 2016, when the correlation between stay green and GY was also strongest. However, multiple linear regression suggested a significant effect of stay green duration on GY even when accounting for heading date, whereas heading date did not have a significant effect on GY (**Supplementary Table 2**). Both heading date and stay green correlated negatively with GPC in both years (2016 and 2017, **Supplementary Table 2**). Thus, it seems that senescence dynamics had a direct effect on GY and GPC in our experiments.

### Visual Senescence Scorings Accurately Track Senescence-Related Processes Affecting Final Grain Yield

**Figure 10:**
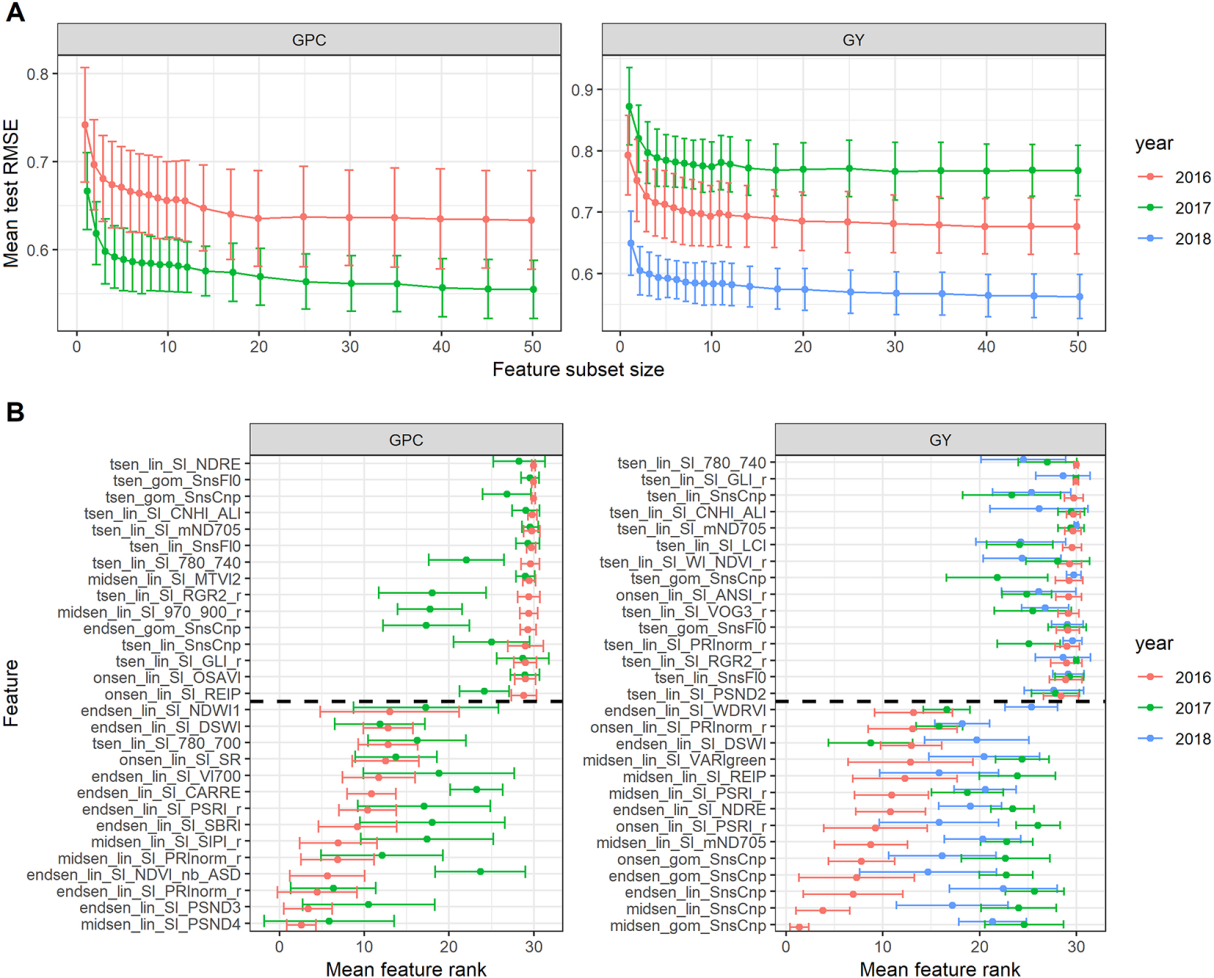
**(A)** Performance of the random forest regression models to predict grain protein concentration (GPC) and grain yield (GY). Mean performance and standard deviation are shown based on 30 resamples of the data for models containing a decreasing number of features selected by recursive feature elimination. **(B)** Feature ranks as determined by recursive feature elimination. Mean feature rank and standard deviation are shown based on 30 resamples of the data for the top and lowest 15 features, separated by the broken line. Features are plotted according to their descending mean rank in the 2016 models.

Given the phenotypic correlations between senescence dynamics parameters and GY and GPC in all 3 years, recursive feature elimination was performed for each trait × year combination. Performance of the models with a given subset size differed across years (**Figure 10A**). Multiple SI improved the prediction accuracy for GY and GPC as compared to single SI (**Figure 10A**). However, after inclusion of two to three features, mean model performance levelled off rapidly. In addition, there was significant variance in model performance estimates and feature ranks across resamples (**Figures 10A, B**). Feature ranks showed lower variance in 2016 for both GY and GPC models, whereas in 2017 and 2018, there was considerable variance across resamples (**Figure 10B**). The most important feature in the 2016 GY model (i.e. midsen derived from the Gompertz model fitted to visual canopy senescence scorings) had an average rank of 1.40 (±0.97), indicating that it was consistently retained as the most predictive feature. For the 2017 GY model, the most important feature had an average rank of 4.10 (±5.57) and for the 2018 GY model, it had an average rank of 5.83 (±4.77), indicating much lower consistency across resamples (data not shown). In the GY model for 2016, features derived from the visual scorings were clearly the most predictive (**Figure 10B**). The features derived from the non-linear fit of visual canopy senescence scorings had lower ranks than the corresponding feature derived from linear interpolations, except for End_sen_, for which the features had almost identical ranks. SI-derived features had much higher mean ranks than scoring derived features. The lowest ranked SI-derived features were derived from the mND705, the PSRI, the NDRE, the REIP, and the VARIgreen. No T_sen_ parameters were among the top 15 features of the GY models for any year, suggesting that the duration of senescence as assessed here did not affect GY in any experiment. Feature ranks were quite unstable across years, particularly for the GY models. In contrast to the GY models, no scoring-derived features were among the most important features in the GPC models. Instead, features derived from the PSND4 and the PRInorm had relatively low mean ranks in both years. This was in strong contrast to the results of the simple regressions, which suggested mainly a negative correlation between the duration of senescence and GPC in 2016 and a negative correlation between visually assessed stay-green and GPC in 2017 (**Table 4**).

## Discussion

### Large Genetic Variability in Senescence Dynamics and Minor Effects on Grain Yield and Grain Protein Concentration

Within-year repeatability of On_sen_, Mid_sen_ and End_sen_ was moderate to high for scoring and SI-derived parameters, which is in line with previous reports (Blake et al., 2007; Lopes and Reynolds, 2012; Crain et al., 2017). Within-year repeatability of T_sen_ derived from some SI was similar, but was nearly zero for visual scorings (**Table 1**). This suggests that the duration and the rate of the senescence process is more accurately estimated using specific SI.

In this study, a positive correlation between the duration of the stay-green phase and GY was observed in all years. However, a strong correlation was found only in the wet season of 2016, whereas in the relatively dry and hot seasons of 2017 and 2018, correlations were weaker (**Table 4**). This is somewhat unexpected, as drought and heat stress are likely to anticipate and accelerate senescence (Gregersen et al., 2013). This could result in source-limited GY and therefore enhance differences in GY between stay-green and early senescing genotypes (Borrás et al., 2004). In 2016, visual senescence scorings were affected by foliar diseases, mainly STB. It seems likely that disease symptoms affected senescence scorings particularly during the late stay-green phase. High levels of STB can reduce GY significantly (*reviewed by* Fones and Gurr, 2015). Thus, differences in STB severity likely contributed to the observed correlation between visually assessed senescence dynamics and GY. Another possibility is that the phenotypic correlation between senescence dynamics and GY in 2016 arose at least in part from pleiotropic effects. Bogard et al. (2011) demonstrated that phenotypic correlations between senescence dynamics and GY were mainly related to differences in flowering date in a doubled haploid mapping population. In our experiments, heading date was significantly correlated with the duration of the stay-green phase, but effects on yield were not statistically significant. This highlights that, in addition to facilitating the investigation of direct effects of secondary trait dynamics on primary traits, the implementation of high throughput phenotyping protocols may equally benefit the elucidation of such pleiotropic effects. A detailed understanding of such interdependencies is paramount to improve genetic crop models and fine tune dynamic traits in breeding (Chenu et al., 2009; Chenu et al., 2017). It should also be understood that such aspects will have to be taken into account when investigating the genetic determinants of senescence dynamics. In the subsequent sections, we discuss the results of different approaches to phenotype senescence as a dynamic trait.

### Spectral Indices Emphasizing Reflectance in the Visible to Near-Infrared Range Accurately Track Canopy Senescence Dynamics

Regular NDVI measurements have been used by several authors to evaluate stay-green, mainly under drought conditions (Lopes and Reynolds, 2012; Christopher et al., 2014; Christopher et al., 2016; Montazeaud et al., 2016; Christopher et al., 2018). In this study, the PSRI gave a better representation of visually recorded canopy senescence dynamics than the NDVI. The dynamics of the SI suggest that the accuracy of the NDVI is not primarily hampered by saturation effects, as it tends to decrease earlier than the PSRI (**Figures 5** and **6**).

At the leaf scale, the PSRI specifically measures changes in pigment composition by comparing the reflectance at 500 nm, which is controlled by the combined absorption of chlorophyll *a*, chlorophyll *b* and carotenoids with absorption at 678 nm, which is controlled by chlorophyll *a* only (Merzlyak et al., 1999). Major changes in pigment composition have been observed for flag leaves of field-grown wheat plants after about 20 days post-anthesis (Lu et al., 2001). These changes in pigment composition coincided with the onset of a steep decrease in total chlorophyll content and thus probably with the onset of chloroplast dismantling, which marks the beginning of senescence (Lu et al., 2001; Havé et al., 2017). Therefore, it appears plausible that the PSRI is indicative of wheat canopy senescence. However, unlike at the leaf scale, R750 changes drastically during senescence at the canopy scale (**Figure 4A**). Thus, PSRI values at the canopy scale are strongly driven by R750. Reflectance in the NIR is dominated by leaf area index among other canopy structure parameters (Jacquemoud et al., 2009). Based on a comparison with RGB images, we hypothesized the PSRI to be less sensitive to variation in canopy structure than the NDVI. The NDVI is highly sensitive to canopy structure, as R800 is one of two constituting wavebands. Canopy structure may change drastically prior to and during senescence. For example, leaf-roll can be induced by water shortage resulting in major canopy structural changes and an increased contribution of soil reflectance that is not necessarily related to senescence. Furthermore, changes in spike geometry are likely to interfere with the retrieval of biochemical information. Both factors strongly affect reflectance in the NIR, while reflectance in the VIS is less affected (Gutierrez et al., 2015). It appears that the inclusion of a second waveband in the VIS stabilized the PSRI against canopy structural effects during early senescence (**Figure 6**).

The relatively low sensitivity of the observed correlations between the PSRI and visual canopy senescence dynamics to shifts in the constituting wavebands suggests that multispectral information is sufficient to obtain accurate estimates of canopy senescence dynamics. This makes the trait amenable to phenotyping using multispectral cameras which can be mounted on unmanned aerial vehicles (Aasen et al., 2018; Aasen and Bolten, 2018). This would greatly facilitate large-scale screenings and frequent measurements. Such large-scale screenings and a high temporal resolution of measurements are likely to be the primary benefits of digital phenotyping of senescence dynamics in the near future.

### Non-Linear Models Outperform PLSR in Tracking Senescence Dynamics, but Are Similarly Environment-Specific

Full-spectrum models improved the inference of visual senescence scorings from spectral data as compared to the best SI, but their power to track senescence dynamics was limited by the extraction of year-specific relationships between reflectance and scorings, and, in the case of PLSR, by their inflexibility to capture non-linear relationships between spectral reflectance and visual scorings. Such non-linearities likely arise from the fact that senescence is a complex process, during which major physiological and structural changes at the leaf and canopy scales occur sequentially or simultaneously with most of them having strong but contrasting effects on the reflectance characteristics of plant canopies. Such changes include chlorophyll degradation and changes in pigment composition, loss of cellular structure, mesophyll breakdown and water loss at the leaf level (Gitelson and Merzlyak, 1994) as well as a reduction in leaf area index and ground cover, changes in leaf and spike geometry, nutrient redistribution to the spikes and water loss at the canopy level.

PLSR failed to accurately track visually observed senescence dynamics in our experiment, and was outperformed by several SI, even when validated on held out samples of the same experiment. Kipp et al. (2014) found no stable relationships between various types of SI and flag leaf color, but reported a good predictive performance of PLSR models. This is not necessarily in contradiction to our observations, since we also found improved prediction of visual senescence scorings when exploiting the full spectrum. However, our objective was not to predict absolute values of greenness, but to track temporal changes throughout the process of senescence and extract parameters that describe these dynamics. Therefore, we scaled both scorings and spectra-derived predictions to a uniform range and only exploited the relative temporal changes (**Figure 2**, upper panel). With this intermediate step, we eliminated initial and terminal differences across genotypes or experimental plots, which can have multiple origins and interfere with the retrieval of dynamics parameters and measures of overall accuracy. The increase in accuracy of cubist compared to PLSR models was paralleled by an increased across-year applicability of the models on average, indicating that the problem of year-specific modeling was not exacerbated by using a more flexible algorithm.

In general, the RMSE of the cubist models was low (<0.7 in 2017 and 2018). We speculate that this is close to the performance ceiling set by the precision of visual scorings. Achieving substantial improvements by further optimizing the models seems therefore unlikely. Rather, more precise ground truth data would be required. Visual scorings are subjective and limited in tracking small changes between assessment time points. SPAD meter or color measurements have been used by other authors (*e.g.* Kipp et al., 2014; Xie et al., 2016). These tend to be more objective, more sensitive to subtle changes and relate more directly to a physiological trait. On the other hand, they sample only a small part of the leaf and are laborious to obtain. Also, senescence typically does not progress uniformly along the leaf, resulting in difficulties to obtain a good average value per plot. Thus, in our opinion, these measurements do not produce better average values per plot than a visual scoring. Furthermore, small gains in precision need to be weighed against the necessity of sampling a sufficiently large genotypic diversity at a high temporal resolution in several years/environments to achieve robust models, as illustrated above.

### Model Transferability Is Strongly Related to Differences in Environmental Conditions

We found major differences in the applicability of models across years. In particular, the dynamics of visual scorings in the 2016 experiments were very poorly predicted by models trained on 2017 and/or 2018 data (**Table 3**). Furthermore, models trained and validated within the 2016 experiment performed poorly compared to the other two years. This could be due to the different measurement protocol applied in 2016. Interestingly, however, models trained using data from 2016 performed well in 2017 and 2018. Therefore, it seems more likely that limited model applicability in 2016 is at least in part a consequence of a larger variability in how progression of senescence affected hyperspectral reflectance across genotypes in this year. In the same experiment and during the same period, major differences were found for STB severity among genotypes and STB was the dominant disease throughout the stay-green phase (*see* Karisto et al., 2018 *for details*). In contrast, in 2017 and 2018 foliar diseases were at very low levels due to dry weather conditions. Several STB severity metrics were found to affect spectral reflectance in 2016, with strong effects particularly in the NIR (*see* Yu et al., 2018 *for details*). We therefore hypothesize that STB altered the temporal evolution of the hyperspectral reflectance signal during the late stay-green and early senescence phases with respect to disease-free plots. Assuming that STB also affected the visual canopy senescence scorings at least during early senescence, this would explain the strong contribution of wavebands in the NIR to models in 2016 (**Figure 8B**). The results of the SI dynamics seem to offer some additional support for this hypothesis. Indeed, the difference in accuracy between the PSRI and the more generic NDVI in tracking visually assessed senescence is relatively small in 2016 as compared to 2017 and 2018 (**Table 2**). This suggests that changes in pigment composition were not much better indicators of senescence in 2016 than was a generic indicator of greenness such as the NDVI. Leaves affected by STB develop necrotic lesions, but do not undergo controlled dismantling of the photosynthetic apparatus resulting in the typical changes in color and in pigment composition probably contributing to the increased performance of the PSRI. Finally, the difference between performance of PLSR and cubist was particularly large for 2016 (**Table 3**). Under the scenario that STB affected overall greenness in the late stay-green and early senescence phase (see above) this pattern is to be expected, since STB should affect the spectral reflectance in a different manner than physiological senescence, which will dominate in later phases, increasing the non-linearity between spectral reflectance and visual senescence scorings through the entire process. We hypothesize that repeated hyperspectral reflectance measurements during late stay-green and throughout senescence might allow to distinguish purely physiological senescence from partly disease-driven loss of green leaf area, and facilitate an indirect assessment of disease resistance in field-grown wheat at high throughput.

### Digital Senescence Phenotyping May Benefit Crop Breeding Primarily Through Increased Temporal Resolution and Throughput of Measurements

Relatively strong linear correlations were observed between senescence dynamics parameters and GY and GPC in 2016 and results from feature selection are most conclusive for this year. Increases in model performance could be observed both for GY and GPC in 2016 when using multiple features and the obtained feature ranks were relatively stable across resamples. For GY, the most important features are either directly derived from visual senescence scorings of the canopy or from SI that were found to predict these scorings well (**Table 2**). Specifically, End_sen_ derived from NDRE is highly correlated to End_sen_ derived from visual scorings (r = 0.73), mND705 was found to be most accurate to predict Mid_sen_ (r = 0.81), followed by the PSRI (r = 0.76). NDRE and mND705 have been developed to improve sensitivity to chlorophyll content with respect to the NDVI (Barnes et al., 2000; Sims and Gamon, 2002). This is achieved primarily by replacing the reflectance in the red by reflectance in the red-edge, which is less prone to saturation at high chlorophyll contents of leaves and vegetation and more robust in presence of leaf or canopy structural effects (Demetriades-Shah et al., 1990; Gitelson and Merzlyak, 1994; Sims and Gamon, 2002).

The low average feature ranks of visual canopy senescence scorings and SI that accurately track these scorings suggest that the dynamics of chlorophyll breakdown was most predictive of GY, and that this trait could be assessed with a high precision using visual scorings or the proposed SI. This can be well explained, as the onset of chlorophyll breakdown marks the onset of remobilization and the end of photo-assimilation, thereby directly affecting source capacity. However, several additional conclusions can be drawn from these findings.

First, it can be concluded that feature selection on time courses of multiple SI resulted in the identification of features most strongly associated with GY and describing a dynamic trait interpretable in terms of plant physiology.

Second, given that no SI-derived feature was more predictive of GY than scoring-derived features, we conclude that potential precision gains in estimating the switch from stay-green to remobilization using hyperspectral high throughput phenotyping techniques rather than visual scorings may be limited. It should be noted, however, that most of the SI used in this study were not developed for use in wheat canopies during senescence, and only few of them have been tested for their applicability during this growth stage (Erdle et al., 2013; Barmeier and Schmidhalter, 2017; Hassan et al., 2018). Significant relationships seem to be maintained during later growth stages, but tend to be unstable across stages (Erdle et al., 2013). Nonetheless, we assume that the selected features summarize a considerable part of the total information contained in hyperspectral measurements during this phase. We further conclude that visual scorings apparently allow assessing a key trait during senescence in a reliable manner. Further research should therefore aim at understanding the factors hampering across-year applicability of otherwise successful full-spectrum models to infer senescence scorings and how these factors can be accounted or corrected for. A method to obtain highly accurate training data of canopy greenness will also be required to achieve good predictive models. Additionally, the lower mean ranks of PSRI-derived features and higher linear correlation coefficients between PSRI-derived features and GY provide additional evidence for the superior precision of the PSRI compared to the NDVI.

Third, in a first step, improvements in precision may be achieved mainly by increasing the temporal resolution of measurements. The higher ranks of features derived from the parametric models are likely the result of the smoothing properties of non-linear model fits, better approximating the gradual nature of the senescence process and reducing the impact of measurement or scoring errors associated with a particular time point on the estimation of dynamics parameters. In addition, parametric models would also allow for the derivation of measures that better separate distinct characteristics of the senescence process. In particular, the derivation of a parameter describing specifically the rate of senescence or any process occurring during senescence, could be highly beneficial to elucidate effects of senescence dynamics on primary traits, particularly GPC and nitrogen use efficiency, but also GY (Gregersen et al., 2008; Wu et al., 2012; Kong et al., 2016; Xie et al., 2016). In contrast, the T_sen_ parameter used here is partly reflected by the other parameters since it was derived by subtracting On_sen_ from End_sen_. It also integrates over the whole process, which may be overly simplistic and may not adequately represent senescence dynamics observed at the leaf or canopy scale (Bogard et al., 2011; Gaju et al., 2014).

Several of the selected features had relatively low linear correlation coefficients whereas some other highly ranked features also had high linear correlation with GY. Thus, it seems that rf extracted some non-linear relationships between features and GY, and these seemed to be more predictive of GY than the linear correlations found for some features. Unfortunately, the final rf model is not interpretable due to its ensemble nature. We chose rf as a base learner for feature selection (i) because it is affected much less by the presence of non-informative predictors and multi-collinearity among predictors than parametrically structured models, (ii) for its capability to capture non-linear relationships between predictors and the response and interactions between predictors which could not be excluded in our case and, most importantly, (iii) precisely because of its ensemble nature that allowed it to produce stable variable importance rankings even in the presence of highly collinear predictors and consequently facilitated the removal of the less important one during subsequent feature elimination steps. We recognize that this may have come at the cost of less-than-optimal performance in the presence of strictly linear relationships between predictors and the response and might, in some cases, have resulted in the extraction of relationships that are difficult to interpret in terms of plant physiology or phenology. However, the fact that the scoring and PSRI-derived features were among the most highly ranked features, while we also found high linear correlations suggests that these weaknesses of the rf algorithm should have impacted the result only marginally.

The above observations could not be confirmed in 2017 and 2018 in spite of the fact that simple linear regressions suggested that visual scorings and corresponding SI were again among the most predictive features (**Table 4**). It seems likely that the overall effect of senescence dynamics on GY and GPC was too weak in 2017 and 2018, which would also explain the increased variability of feature ranks across resamples. In the presence of small effects and under the hypothesis that the correlations between features and responses are close to linear, the results of linear regressions may be more reliable.

Finally, it should be noted that our analysis was based on the observation of a phenotypic correlation between senescence dynamics parameters and primary traits. We did not observe a significant effect of heading date on GY. Nevertheless, it cannot be excluded with certainty that this phenotypic correlation arose primarily as a result of pleiotropic effects, and this might have affected our conclusions. Subjecting genotypes to very harsh conditions post-anthesis is likely to accentuate direct effects of senescence dynamics on primary traits, enabling a more precise evaluation of the potential benefits of a high spectral resolution during late development.

## Conclusions

Using existing variability in senescence dynamics for wheat improvement requires intensive field-testing of large populations in contrasting environments. We hypothesized that repeated spectral reflectance measurements may facilitate an accurate assessment of this developmental phase at high throughput. Our results show that time series of the PSRI accurately track visually observed canopy senescence dynamics across a large number of genotypes and under varying environmental conditions. When a substantial effect of senescence dynamics on GY was present, correlations between scoring-derived and PSRI-derived senescence dynamics parameters and GY were very similar. We therefore conclude that visual scorings could be replaced by PSRI measurements without a significant loss in precision. On the other hand, the high spectral resolution of measurements did not confer significant advantages over visual scorings or measurements of a single spectral index in our experiment. This is encouraging for the breeding and plant-phenotyping community, since it implies that senescence dynamics may be accurately tracked using less sophisticated and potentially cheaper spectral sensors. Thus, we conclude that digital senescence phenotyping will benefit wheat breeding through an increased temporal resolution and high throughput of measurements.

## Data Availability Statement

Data sets generated and analyzed in this study are available from the ETH Zürich publications and research data repository (https://www.research-collection.ethz.ch). Experimental data supporting the conclusions of this article can be downloaded here: https://doi.org/10.3929/ethz-b-000365618.

All analysis scripts required to reproduce the results published in this article are publicly available.

An archived version can be retrieved from: https://doi.org/10.5905/ethz-1007-227.

Development repository: https://github.com/and-jonas/Andereggetal2019.

Programming language: R. License: GNU General Public License, version 3 (GPL-3.0).

## Author Contributions

AH, AW, and KY designed and planned the experiment. KY and JA performed spectral reflectance measurements as supervised by FL and HA. JA performed visual scorings, analyzed the data, and drafted the manuscript. AH, KY, and HA supervised the project. All authors contributed to discussion of data and to writing and revision of the manuscript.

## Funding

The project was partially funded by the Swiss Federal Office of Agriculture (FOAG).

## Conflict of Interest

The authors declare that the research was conducted in the absence of any commercial or financial relationships that could be construed as a potential conflict of interest.
