# Supplementary material for: Spectral Vegetation Indices to Track Senescence Dynamics in Diverse Wheat Germplasm
Source: Front Plant Sci. 2020 Jan 28;10:1749. doi: 10.3389/fpls.2019.01749 (PMC6997566; doi:10.3389/fpls.2019.01749)
Supplement: Supplementary file 1 [file DataSheet_1.docx]

Supplementary Material

# Supplementary Methods

**Calculation of spectral indices and extraction of dynamics parameters**

Spectral regions comprising the wavelengths from 1350 nm to 1475 nm, from 1781 nm to 1990 nm and from 2400 nm to 2500 nm were removed because of the very low signal-to-noise ratio resulting from high atmospheric absorption. Spectra were then averaged for each experimental plot and 100 published spectral indices (SI) sensitive to chlorophyll content, water content, N content and total above ground N, leaf area index, green biomass and vegetation cover, and pigment changes and plant senescence were computed according to the formulae reported in Table S1 (Figure 1, [1]). Values of SI were scaled to range from 0 to 10, representing the minimum and maximum values recorded during the assessment period, respectively. Scaled values of these SI were then fitted against thermal time after heading using linear interpolation and dynamics parameters were extracted as was done for visual scorings (Figure 1, [2]). Parametric models were not used for SI as different SI exhibited distinct temporal patterns and would have required the use of SI-specific models (*compare* Figure 5).

**Spectral index subset selection**

We then performed unsupervised subset selection to reduce multi-collinearity of the dataset using several filtering criteria (Figure 1, [3]). Only SI that showed a monotonous or near-monotonous decrease during the assessment period for most experimental plots were retained for further analysis. Repeatability (w^2^) of the senescence dynamics parameters derived from the selected SI was then calculated based on data obtained in 2016. Only parameters with w^2^ similar to those observed for the visual scorings were retained (w^2^ > 0.6 for On_sen_, w^2^ > 0.7 for Mid_sen_, w^2^ > 0.8 for End_sen_ and w^2^ > 0.5 for T_sen_). Finally, highly correlated parameters were iteratively removed by calculating a correlation matrix based on three years data and discarding one of two parameters with a pair-wise correlation coefficient > 0.96. Parameters were selected preferring narrow-band SI over broad-band SI, SI with a specific physiological interpretation over more generic SI and SI developed specifically for use in wheat or barley canopies. Additionally, the smoothness of SI values over time was evaluated graphically and used as an additional selection criterion. This selection procedure resulted in a set of 83 SI-derived senescence dynamics parameters based on 51 distinct SI with pair-wise correlation coefficients r < 0.96.

**Development and validation of full-spectrum models to infer visual senescence scorings**

As an alternative to published SI, two multivariate modelling techniques were used to infer senescence scorings from spectral data (Figure 1, [4]) : (1) Partial Least Squares Regression (PLSR), which has been used extensively for analyzing field measured hyperspectral reflectance data (*see* Wold et al., 2001 *for details*) and (2) cubist regression, which substantially builds on the M5 model tree (Quinlan, 1992), extending it by incorporating a boosting-like procedure referred to as *committees*. The latter algorithm is more flexible since it does not require the user to specify the nature of the relationship between predictors and the response and is able to capture more complex non-linear relationships (Kuhn and Johnson, 2013). The R packages *‘pls’* V2.7.0 (Mevik et al., 2018) and *‘Cubist’* (Kuhn et al., 2018) were used for the analysis. The hyperparameters of each model were tuned using 10-fold cross-validation, which was repeated 5 times in the case of PLSR, using the R package ‘*caret*’ V6.0.80 (Kuhn, 2008). As the main interest lies on the capability of full-spectrum models to represent the entire process of senescence, average RMSE for 10 different random upsamples of the test data were calculated, with each upsample containing all possible scoring values an equal number of times, i.e. exactly the number of times of the most frequent observation. This was achieved by random sampling with replacement.

A common issue with full-spectrum models is limited applicability on data of experiments not contained in the model training process (*see e.g.* Øvergaard et al., 2013). We hypothesized that this problem should be less pronounced in our case, as senescence results in major changes of canopy reflectance that should be similar across years. Still, to maximize model across-year applicability, we evaluated different types of input data: (1) raw reflectance spectra, (2) smoothed reflectance spectra, (3) first derivative of smoothed reflectance spectra, (4) continuum removed smoothed reflectance spectra and (5) raw reflectance spectra limited to the spectral range between 500 nm and 700 nm, as done by Kipp et al. (2014). Smoothing of raw spectra and calculation of derivatives was done using the Savitzky-Golay smoothing filter (Savitzky and Golay, 1964) with a window size of 11 spectral bands and a third order polynomial. Continuum removal (Clark and Roush, 1984) was done using linear interpolation between extrema and subtraction for normalization. Spectra pre-preprocessing was done using functions of the R package ‘*prospectr*’ V0.1.3 (Ramirez-Lopez and Stevens, 2014). Each type of input data was tested without pre-treatment and with mean-centering and scaling to unit variance of all predictors. Finally, feature selection was conducted following the procedure described in the next section to identify the most important wavelengths for each year. Model evaluation was done within experiments (years) as well as across experiments to estimate the robustness of the developed models. Validation datasets were randomly up-sampled to obtain an estimate of model performance across the whole process of senescence.

**Supervised feature selection**

Supervised feature selection was performed by recursive feature elimination using a nested cross-validation approach. Cubist was used as a base-learner for full-spectrum models to infer senescence scorings, while rf was used as a base-learner for selection of scoring- and SI-based senescence dynamics parameters. The ‘*ranger*’ implementation (Wright and Ziegler, 2017) of the original rf algorithm (Breiman, 2001) was used (R package *‘ranger’* V0.10.1). The *mtry* hyperparameter was tuned using 10-fold cross-validation, while variance reduction was defined as a split criterion, the minimal node size was set to five samples and the number of trees was set to 10,000, resulting in stable feature importance rankings for given resamples. Feature importance was calculated based on permutation. In order to obtain a robust ranking of features and measure of model performance, the data set was resampled 30 times into training and test sets with an 80:20 split. For each resample, a rf was trained using all features, as described above. Then, from the set of original features, those with the lowest importance values were removed iteratively in 29 decreasing steps until only one feature remained in the model. After each iteration, feature importance was re-calculated due to remaining multi-collinearity in the feature set and performance was assessed on the corresponding test set. The results of the 30 resamples were then aggregated to obtain a performance profile over the feature subset sizes and robust feature importance rankings. For full-spectrum models, the procedure was identical. Here, the varImp() function of the R package *‘caret’* was used to extract variable importance in each iteration. The returned measure is a linear combination of the usage in rule conditions and the model. For more details, we refer to Kuhn and Johnson(2013).

# Supplementary Tables

**Supplementary Table 1.** Hyperspectral vegetation indices used in this study to track senescence dynamics.

| **Index** | **Application** | **Formula** | **Reference** |
| --- | --- | --- | --- |
| ANTH | Anthocyanin | R760to800*(1/R540to560-1/R690to710) | (Gitelson et al., 2006) |
| ARI | Anthocyanin | 1/(R549to551*100)-1/(R699to701*100) | (Gitelson et al., 2001) |
| ARVI | LAI/vegetation fraction/green biomass | (R845to885-(R845to885-R460to480+R845to885))/(R845to885+(R635to685-R460to480+R635to685)) | (Kaufman and Tanre, 1992) |
| CAI | Plant litter / crop residues | 0.5*(R2019to2021*100+R2219to2221*100)-R2099to2101*100 | (Nagler et al., 2000) |
| CARG | Car | R760to800*(1/R510to520-1/R540to560) | (Gitelson et al., 2006) |
| CARRE | Car | R760to800*(1/R510to520-1/R690to710) | (Gitelson et al., 2006) |
| CHLG | Chl | R760to800/R540to560 | (Gitelson et al., 2006) |
| CHLRE | Chl | R760to800/R690to720-1 | (Gitelson et al., 2006) |
| CIG | Chl / LAI | R750to800/R520to585-1 | (Gitelson et al., 2003) |
| CIRE | Chl / LAI | R750to800/R695to740-1 | (Gitelson et al., 2003) |
| CLSI | Disease discrimination | (R697to699-R569to571)/(R697to699+R569to571)-R733to735 | (Mahlein et al., 2013) |
| CNHI | Senescence | (R8455to890-R1200to1300)*(R775to805+R630to690))/( R8455to890+ R1200to1300)*(R775to805- R630to690)) | (Pimstein et al., 2009) |
| CRI1 | Car | 1/R506to514-1/R535to565 | (Gitelson et al., 2002b) |
| CRI2 | Car | 1/R506to514-1/R693to707 | (Gitelson et al., 2002b) |
| DCNI | Plant N concentration | (R717to723-R697to703)/(R697to703-R667to673)/( R717to723- R667to673+0.03) | (Chen et al., 2010) |
| DSWI | Disease discrimination | (R795to805+R543to553)/R1654to1664+R676to686) | (Apan et al., 2004) |
| EVI | LAI / Vegetation fraction | 2.5*(R841to876-R620to670)/(R841to876+6*R620to670-7.5*R459to479+1) | (Huete et al., 2002) |
| FII | Flowering | (R470to480-R360to370)/(R470to480+R360to370) | (Stuckens et al., 2011) |
| GBNDVI | N concentration / LAI | (R567to579-R438to442)/(R567to579+R438to442) | (Hansen and Schjoerring, 2003) |
| GLI | Vegetation cover / Chl | (2*R580to610-R580to660-R400to520)/(2*R580to610+ R580to660+ R400to520) | (Louhaichi et al., 2001) |
| GM | Chl | R841to876/R545to565 - 1 | (Gitelson et al., 2005) |
| HI | Disease discrimination | (R533to535-R697to698)/(R533to535+R697to699)-1/2*R703to705 | (Mahlein et al., 2013) |
| LCI | Chl / Senescence | (R849to851+R709to711)/(R849to851+R679to681) | (Datt, 1999) |
| LCI2 | Chl / Senescence | (R849to851-R709to711)/(R849to851+R679to681) | (Datt, 1999) |
| LWI | Leaf water content / EWT | R1250to1350/R1400to1500 | (Seelig et al., 2008) |
| LWVI1 | Leaf water | (R1089to1099-R888to898)/(R1089to1099+R888to898) | (Galvão et al., 2005) |
| LWVI2 | Leaf water | (R1089to1099-R1201to1209)/( R1089to1099+R1201to1209) | (Galvão et al., 2005) |
| MCARI | LAI | {1.5*[2.5*(R797to803 - R676to673)-1.3*(R797to803 – R547to553)]}/  sqrt[(2* R797to803 + 1)^2-(6* R797to803-5*sqrt( R676to673)) - 0.5] | (Haboudane et al., 2004) |
| MCARI/MTVI2 | Leaf N concentration (SPAD) | MCARI/MTVI2 | (Eitel et al., 2007) |
| MCARI/OSAVI | Chl | MCARI/OSAVI | (Wu et al., 2008) |
| mND705 | Chl | (R749to751-R704to706)/(R749to751+ R704to706-2*R444to446) | (Sims and Gamon, 2002) |
| MSAVI | Green cover / LAI | 0.5*(2*R790to890+1-sqrt((2*R790to890+1)^2-8*(R790to890-R610to690))) | (Qi et al., 1994) |
| MSR_rev_ | Chl | [(R750/R705) + 1]/sqrt[(R750/R705) + 1] | (Wu et al., 2008) |
| MTCI | Chl | (R751to757-R705to713)/(R705to713-R678to684) | (Dash and Curran, 2004) |
| MTVI1 | LAI | 1.2*(1.2*(R797to803-R547to553)-2.5*(R667to673-R547to553)) | (Haboudane et al., 2004) |
| MTVI2 | LAI | {1.5*[1.2*(R797to803+R547to553)+2.5*(R667toR773+R547to553)]}/  sqrt[(2*R797to803+1)^2 + (6*R797to803+5*sqrt(R667toR773))+0.5] | (Haboudane et al., 2004) |
| NDLI | foliar/bulk canopy lignin (senescence) and N  concentration | (log(1/R1750to1758)-log(1/ R1676to1684))/(log(1/ R1750to1758)+log(1/ R1676to1684)) | (Serrano et al., 2002) |
| NDMI | Vegetation water content | (R760to900-R1550to1750)/(R760to900+R1550to1750) | (Hardisky et al., 1984) |
| NDNI | Foliar/bulk canopy N concentration | [log(1/R1506to1514) - log(1/R1676to1684)] / [log(1/ R1506to1514) +  log(1/ R1676to1684)] | (Eitel et al., 2007) |
| NDRE | Crop cover / Chl / water / N stress | (R786to794-R716to724)/(R786to794+R716to724) | (Barnes et al., 2000) |
| NDREI | Chl-a / Senescence | (R749to751-R704to706)/(R749to751+R704to706) | (Gitelson and Merzlyak, 1994) |
| NDSVI | Residues / Senescence | (R1550to1750-R630to690)/(R1550to1750+R630to690) | (Qi et al., 2002) |
| NDTI | Tillage (residue cover) / soil plain | (R1550to1750-R2080to2350)/( R1550to1750+R2080to2350) | (Deventer et al., 1997) |
| NDVI | LAI / Vegetation fraction / Green biomass | (R799to801-R669to671)/(R799to801+R669to671) | (Rouse et al., 1974) |
| NDWI | Water content | (R856to864-R1236to1244)/(R856to864+R1236to1244) | (Gao, 1996) |
| NDWI1650 | Water content | (R770to910 – R1550to1750)/( R770to910 + R1550to1750) | (Clay et al., 2006) |
| NDWI2130 | Water content | (R841to876-R2105to2155)/(R841to876+R2105to2155) | (Chen et al., 2005) |
| NGRDI | Biomass / Water content / Chl | (R520to600 – R630to690)/(R520to600 + R630to690) | (Tucker, 1979) |
| NHI | Heading of wheat | (R1098to1102-R1198to1202)/( R1098to1102+R1198to1202) | (Pimstein et al., 2009) |
| NPCI | Chl/Car ratio | (R678to682-R428to432)/(R678to682+R428to432) | (Peñuelas et al., 1994) |
| OCAR | Chl | R629to631/R679to681 | (Schlemmer et al., 2005) |
| OSAVI | Vegetation cover / LAI / Chl | (1+0.16)*(R797to803-R667to673)/(R797to803+R667to673+0.16) | (Rondeaux et al., 1996) |
| PBI | Chl / N | R806to814/R556to564 | (Rao et al., 2008) |
| PMI | Disease discrimination | (R519to521-R583to583)/(R519to521+R583to583)+R723to725 | (Mahlein et al., 2013) |
| PRInorm | Xanthophyll / Car / Canopy leaf area | [(R570-R531)/(R570+R531)] / {[(R800-R670) /sqrt(R800+R670)]*(R700/R670)} | (Zarco-Tejada et al., 2013) |
| PSND1 | Chl / Car | (R799to801-R674to676)/( R799to801+R674to676) | (Blackburn, 1998) |
| PSND2 | Chl / Car | (R799to801-R649to651)/(R799to801+R649to651) | (Blackburn, 1998) |
| PSND3 | Chl / Car | (R799to801-R499to501)/(R799to801+R499to501) | (Blackburn, 1998) |
| PSND4 | Chl / Car | (R799to801-R469to471)/(R799to801+ R469to471) | (Blackburn, 1998) |
| PSRI | Pigment changes / Senescence | (R677to679-R499to501)/R749to751 | (Merzlyak et al., 1999) |
| PSSR1 | Chl/Car | R799to801/R674to676 | (Blackburn, 1998) |
| PSSR2 | Chl/Car | R799to801/R649to651 | (Blackburn, 1998) |
| PSSR3 | Chl/Car | R799to801/R499to501 | (Blackburn, 1998) |
| R1200 | Leaf water content | (2*R1180to1220)/(R1090to1110+R1265to1285) | (Pu, 2011) |
| R760/R730 | Dry matter / Total aerial N | R759to761/R729to731 | (Mistele and Schmidhalter, 2010b) |
| R780/R550 | Dry matter / Total aerial N | R779to781/R549to551 | (Takebe et al., 1990) |
| R780/R700 | Dry matter / Total aerial N | R779to781/R699to701 | (Mistele and Schmidhalter, 2010a) |
| R780/R740 | Dry matter / Total aerial N | R779to781/R739to741 | (Mistele and Schmidhalter, 2010a) |
| R970/R900 | Dry matter / Total aerial N | R969to971/R899to901 | (Mistele and Schmidhalter, 2010b) |
| R975 | Water content | (2*R960to990)/(R920to940 + R1090to1110) | (Pu et al., 2003) |
| REIP | Dry matter / Total aerial N | 700+40*[(R669to671+R779to781)/2-R699to701]/(R739to741-R699to701) | (Guyot et al., 1988) |
| RGR | Anthocyanin / LAI | R682toR684/R509toR511 | (Gamon and Surfus, 1999) |
| RGR2 | Anthocyanin / LAI | R600to699/R500to599 | (Gamon and Surfus, 1999) |
| RRDI_Red-edge_ | Chl | (R744to746-R739to741)/(R739to741-R699to701) | (Yu et al., 2014) |
| RVI2 | Leaf N accumulation (g N m^-2^) | R805to815/R655to665 | (Xue et al., 2004) |
| SAVI | LAI / vegetation fraction / (green biomass) | (1.5)*(R857to863-R667to673)/(R857to863+R667to673+0.5) | (Huete, 1988) |
| SBRI | Disease discrimination | R569to571-R512to514)/(R569to571+R512to514)-1/2*R703to705 | (Mahlein et al., 2013) |
| SGR | Green vegetation cover | $\sum R500to599$ | (Fuentes et al., 2001) |
| SINDRI | Fraction residue cover | (R2185to2225-R2235to2285)/( R2185to2225+R2235to2285) | (Serbin et al., 2009) |
| SIPI | Chl / Car / (Senescence) | R799to801-R444to446)/(R799to801-R679to681) | (Penuelas et al., 1995) |
| SIPI | Chl, Car / LUE / Senescence | (R799to801−R444to446)/(R799to801−R679to681) | (Penuelas et al., 1995) |
| SIWSI | Water stress | (R841to876-R1628to1652)/( R841to876+R1628to1652) | (Fensholt and Sandholt, 2003) |
| SLAIDI1 | LAI | 5*(R1049to1051-R1249to1251)/(R1049to1051+R1249to1251) | (Delalieux et al., 2008) |
| SLAIDI2 | LAI | 40*R1554to1556*(R1049to1051-R1249to1251)/(R1049to1051+R1249to1251) | (Delalieux et al., 2008) |
| SRWI | Water content | R841to876/R1230to1250 | (Zarco-Tejada et al., 2003) |
| TCARI | Chl | 3*((R697to703-R667to673)-0.2*( R697to703-R547to553)*( R697to703/ R667to673)) | (Haboudane et al., 2002) |
| TCARI/OSAVI | Chl | TCARI/OSAVI | (Haboudane et al., 2002) |
| TGI | Chl | -0.5*(190*(R666to674-R546toR554)-120*(R666to674-R476to484)) | (Hunt et al., 2011) |
| VARIgreen | Vegetation fraction | (R546to556-R620to670)/(R546to556+R620to670-R459to479) | (Gitelson et al., 2002a) |
| VI700 | Vegetation fraction | (R700to710-R620to670)/(R700to710+R620to670) | (Gitelson et al., 2002a) |
| VIgreen | Vegetation fraction | (R546to556-R620to670)/(R546to556+R620to670) | (Gitelson et al., 2002a) |
| VIopt | N in crop (kg N ha^-1^) | (1+0.45)*((R760to900)*2+1)/(R630to690+0.45) | (Reyniers et al., 2006) |
| VOG1 | Chl | R739to741/R719to721 | (Vogelmann et al., 1993) |
| VOG2 | Chl | (R733to735-R746to748)/(R714to716+R725toR727) | (Vogelmann et al., 1993) |
| VOG3 | Chl | R733to735-R746to748)/(R714to716+R719to721) | (Vogelmann et al., 1993) |
| WDRVI | LAI / Vegetation fraction / (green biomass) | (0.1*R750to1000-R580to680)/(0.1*R750to1000+R580to680) | (Gitelson, 2004) |
| WI | Leaf water content | R896to904/R966to974 | (Peñuelas et al., 1993) |
| WI/NDVI | Plant water content | (R896to904/R966to974)/((R796to804-R676to684)/(R796to804-R676to684)) | (Penuelas et al., 1997) |
| YCAR | Chl | R599to601/R679to681 | (Schlemmer et al., 2005) |

**Supplementary Table 2.** Least squares coefficient estimates associated with the regression of grain yield (GY) or grain protein concentration (GPC) in 2016-2018 onto heading (in GDD after sowing) and midpoint of senescence (midsen, in GDD after heading) as assessed visually. In a first step, the interaction term was included in the model, but was subsequently dropped, as the analysis did not suggest the presence of a statistically significant interaction. Bold numbers highlight significant p values at the significance threshold of 0.05.

|  | Trait | Coefficient | Std.error | t-statistic | p-value |
| --- | --- | --- | --- | --- | --- |
| GY 2016 | heading | -1.00E-02 | 1.24E-02 | -0.8 | 0.42 |
|  | midsen | -1.45E-02 | 2.71E-02 | -0.54 | 0.59 |
|  | heading:midsen | 1.30E-05 | 1.76E-05 | 0.74 | 0.46 |
| GY 2017 | heading | -2.94E-03 | 1.42E-02 | -0.21 | 0.83 |
|  | midsen | -5.51E-05 | 2.33E-02 | 0.00 | 1.00 |
|  | heading:midsen | 2.83E-06 | 2.06E-05 | 0.14 | 0.89 |
| GY 2018 | heading | 8.82E-03 | 1.09E-02 | 1.54 | 0.12 |
|  | midsen | 1.78E-02 | 2.36E-02 | 1.73 | 0.08 |
|  | heading:midsen | -1.07E-05 | 1.53E-05 | -1.89 | 0.06 |
| GPC 2016 | heading | 1.68E-02 | 1.09E-02 | 1.54 | 0.12 |
|  | midsen | 4.08E-02 | 2.36E-02 | 1.73 | 0.08 |
|  | heading:midsen | -2.88E-05 | 1.53E-05 | -1.89 | 0.06 |
| GPC 2017 | heading | -2.40E-02 | 1.13E-02 | -2.12 | **0.03** |
|  | midsen | -4.10E-02 | 1.86E-02 | -2.20 | **0.03** |
|  | heading:midsen | 3.16E-05 | 1.64E-05 | 1.93 | 0.05 |
| GY 2016 | heading | -7.92E-04 | 1.01E-03 | -0.79 | 0.43 |
|  | midsen | 5.37E-03 | 9.34E-04 | 5.75 | **0.00** |
| GY 2017 | heading | -9.91E-04 | 9.23E-04 | -1.07 | 0.28 |
|  | midsen | 3.15E-03 | 1.12E-03 | 2.82 | **0.01** |
| GY 2018 | heading | 1.77E-03 | 7.10E-04 | 2.50 | **0.01** |
|  | midsen | 2.86E-03 | 8.38E-04 | 3.41 | **0.00** |
| GPC 2016 | heading | -3.69E-03 | 8.80E-04 | -4.19 | **0.00** |
|  | midsen | -3.57E-03 | 8.16E-04 | -4.37 | **0.00** |
| GPC 2017 | heading | -2.24E-03 | 7.38E-04 | -3.03 | **0.00** |
|  | midsen | -5.23E-03 | 8.93E-04 | -5.86 | **0.00** |

Apan, A., Held, A., Phinn, S., Markley, J., 2004. Detecting sugarcane ‘orange rust’ disease using EO-1 Hyperion hyperspectral imagery. International Journal of Remote Sensing 25, 489–498. https://doi.org/10.1080/01431160310001618031

Barnes, E.M., Clarke, T.R., Richards, S.E., Colaizzi, P.D., Haberland, J., Kostrzewski, M., Waller, P., Choi, C., Riley, E., Thompson, T., Lascano, R.J., Li, H., Moran, M.S., 2000. Coincident detection of crop water stress, nitrogen status and canopy density using ground-based multispectral data. Proceedings of the 5th International Conference on Precision Agriculture, Bloomington, Minnesota, USA, 16-19 July, 2000 1–15.

Blackburn, G.A., 1998. Spectral indices for estimating photosynthetic pigment concentrations: A test using senescent tree leaves. International Journal of Remote Sensing 19, 657–675. https://doi.org/10.1080/014311698215919

Breiman, L., 2001. Random Forests. Machine Learning 45, 5–32. https://doi.org/10.1023/A:1010933404324

Chen, D., Huang, J., Jackson, T.J., 2005. Vegetation water content estimation for corn and soybeans using spectral indices derived from MODIS near- and short-wave infrared bands. Remote Sensing of Environment 98, 225–236. https://doi.org/10.1016/j.rse.2005.07.008

Chen, P., Haboudane, D., Tremblay, N., Wang, J., Vigneault, P., Li, B., 2010. New spectral indicator assessing the efficiency of crop nitrogen treatment in corn and wheat. Remote Sensing of Environment 114, 1987–1997. https://doi.org/10.1016/j.rse.2010.04.006

Clark, R.N., Roush, T.L., 1984. Reflectance spectroscopy: Quantitative analysis techniques for remote sensing applications. Journal of Geophysical Research: Solid Earth 89, 6329–6340. https://doi.org/10.1029/JB089iB07p06329

Clay, D.E., Kim, K.-I., Chang, J., Clay, S.A., Dalsted, K., 2006. Characterizing Water and Nitrogen Stress in Corn Using Remote Sensing. Agronomy Journal 98, 579–587. https://doi.org/10.2134/agronj2005.0204

Dash, J., Curran, P.J., 2004. The MERIS terrestrial chlorophyll index. International Journal of Remote Sensing 25, 5403–5413. https://doi.org/10.1080/0143116042000274015

Datt, B., 1999. A New Reflectance Index for Remote Sensing of Chlorophyll Content in Higher Plants: Tests using Eucalyptus Leaves. Journal of Plant Physiology 154, 30–36. https://doi.org/10.1016/S0176-1617(99)80314-9

Delalieux, S., Somers, B., Hereijgers, S., Verstraeten, W.W., Keulemans, W., Coppin, P., 2008. A near-infrared narrow-waveband ratio to determine Leaf Area Index in orchards. Remote Sensing of Environment 112, 3762–3772. https://doi.org/10.1016/j.rse.2008.05.003

Deventer, A.P. van (Geogroep I., Ward, A.D., Gowda, P.H., Lyon, J.G., 1997. Using thematic mapper data to identify contrasting soil plains and tillage practices. Photogrammetric engineering and remote sensing (USA).

Eitel, J.U.H., Long, D.S., Gessler, P.E., Smith, A.M.S., 2007. Using in‐situ measurements to evaluate the new RapidEye^TM^ satellite series for prediction of wheat nitrogen status. International Journal of Remote Sensing 28, 4183–4190. https://doi.org/10.1080/01431160701422213

Fensholt, R., Sandholt, I., 2003. Derivation of a shortwave infrared water stress index from MODIS near- and shortwave infrared data in a semiarid environment. Remote Sensing of Environment 87, 111–121. https://doi.org/10.1016/j.rse.2003.07.002

Fuentes, D.A., Gamon, J.A., Qiu, H., Sims, D.A., Roberts, D.A., 2001. Mapping Canadian boreal forest vegetation using pigment and water absorption features derived from the AVIRIS sensor. Journal of Geophysical Research: Atmospheres 106, 33565–33577. https://doi.org/10.1029/2001JD900110

Galvão, L.S., Formaggio, A.R., Tisot, D.A., 2005. Discrimination of sugarcane varieties in Southeastern Brazil with EO-1 Hyperion data. Remote Sensing of Environment 94, 523–534. https://doi.org/10.1016/j.rse.2004.11.012

Gamon, J.A., Surfus, J.S., 1999. Assessing Leaf Pigment Content and Activity with a Reflectometer. The New Phytologist 143, 105–117.

Gao, B., 1996. NDWI—A normalized difference water index for remote sensing of vegetation liquid water from space. Remote Sensing of Environment 58, 257–266. https://doi.org/10.1016/S0034-4257(96)00067-3

Gitelson, A., Merzlyak, M.N., 1994. Spectral Reflectance Changes Associated with Autumn Senescence of Aesculus hippocastanum L. and Acer platanoides L. Leaves. Spectral Features and Relation to Chlorophyll Estimation. Journal of Plant Physiology 143, 286–292. https://doi.org/10.1016/S0176-1617(11)81633-0

Gitelson, A.A., 2004. Wide Dynamic Range Vegetation Index for Remote Quantification of Biophysical Characteristics of Vegetation. Journal of Plant Physiology 161, 165–173. https://doi.org/10.1078/0176-1617-01176

Gitelson, A.A., Gritz †, Y., Merzlyak, M.N., 2003. Relationships between leaf chlorophyll content and spectral reflectance and algorithms for non-destructive chlorophyll assessment in higher plant leaves. Journal of Plant Physiology 160, 271–282. https://doi.org/10.1078/0176-1617-00887

Gitelson, A.A., Kaufman, Y.J., Stark, R., Rundquist, D., 2002a. Novel algorithms for remote estimation of vegetation fraction. Remote Sensing of Environment 80, 76–87. https://doi.org/10.1016/S0034-4257(01)00289-9

Gitelson, A.A., Keydan, G.P., Merzlyak, M.N., 2006. Three-band model for noninvasive estimation of chlorophyll, carotenoids, and anthocyanin contents in higher plant leaves. Geophysical Research Letters 33. https://doi.org/10.1029/2006GL026457

Gitelson, A.A., Merzlyak, M.N., Chivkunova, O.B., 2001. Optical Properties and Nondestructive Estimation of Anthocyanin Content in Plant Leaves¶. Photochemistry and Photobiology 74, 38–45. https://doi.org/10.1562/0031-8655(2001)0740038OPANEO2.0.CO2

Gitelson, A.A., Viña, A., Ciganda, V., Rundquist, D.C., Arkebauer, T.J., 2005. Remote estimation of canopy chlorophyll content in crops. Geophysical Research Letters 32. https://doi.org/10.1029/2005GL022688

Gitelson, A.A., Zur, Y., Chivkunova, O.B., Merzlyak, M.N., 2002b. Assessing Carotenoid Content in Plant Leaves with Reflectance Spectroscopy¶. Photochemistry and Photobiology 75, 272–281. https://doi.org/10.1562/0031-8655(2002)0750272ACCIPL2.0.CO2

Guyot, G., Baret, F., Major, D.J., 1988. High Spectral Resolution: Determination of spectral shifts between the red and near infrared. The International Archives of Photogrammetry, Remote Sensing and Spatial Information Sciences 750–760.

Haboudane, D., Miller, J.R., Pattey, E., Zarco-Tejada, P.J., Strachan, I.B., 2004. Hyperspectral vegetation indices and novel algorithms for predicting green LAI of crop canopies: Modeling and validation in the context of precision agriculture. Remote Sensing of Environment 90, 337–352. https://doi.org/10.1016/j.rse.2003.12.013

Haboudane, D., Miller, J.R., Tremblay, N., Zarco-Tejada, P.J., Dextraze, L., 2002. Integrated narrow-band vegetation indices for prediction of crop chlorophyll content for application to precision agriculture. Remote Sensing of Environment 81, 416–426. https://doi.org/10.1016/S0034-4257(02)00018-4

Hansen, P.M., Schjoerring, J.K., 2003. Reflectance measurement of canopy biomass and nitrogen status in wheat crops using normalized difference vegetation indices and partial least squares regression. Remote Sensing of Environment 86, 542–553. https://doi.org/10.1016/S0034-4257(03)00131-7

Hardisky, M.A., Daiber, F.C., Roman, C.T., Klemas, V., 1984. Remote sensing of biomass and annual net aerial primary productivity of a salt marsh. Remote Sensing of Environment 16, 91–106. https://doi.org/10.1016/0034-4257(84)90055-5

Huete, A., Didan, K., Miura, T., Rodriguez, E.P., Gao, X., Ferreira, L.G., 2002. Overview of the radiometric and biophysical performance of the MODIS vegetation indices. Remote Sensing of Environment, The Moderate Resolution Imaging Spectroradiometer (MODIS): a new generation of Land Surface Monitoring 83, 195–213. https://doi.org/10.1016/S0034-4257(02)00096-2

Huete, A.R., 1988. A soil-adjusted vegetation index (SAVI). Remote Sensing of Environment 25, 295–309. https://doi.org/10.1016/0034-4257(88)90106-X

Hunt, E.R.J., Daughtry, C.S.T., Eitel, J.U.H., Long, D.S., 2011. Remote Sensing Leaf Chlorophyll Content Using a Visible Band Index. Agronomy journal.

Kaufman, Y.J., Tanre, D., 1992. Atmospherically resistant vegetation index (ARVI) for EOS-MODIS. IEEE Transactions on Geoscience and Remote Sensing 30, 261–270. https://doi.org/10.1109/36.134076

Kipp, S., Mistele, B., Schmidhalter, U., 2014. Identification of stay-green and early senescence phenotypes in high-yielding winter wheat, and their relationship to grain yield and grain protein concentration using high-throughput phenotyping techniques. Funct. Plant Biol. 41, 227–235.

Kuhn, M., 2008. Building Predictive Models in *R* Using the **caret** Package. Journal of Statistical Software 28. https://doi.org/10.18637/jss.v028.i05

Kuhn, M., Johnson, K., 2013. Applied Predictive Modeling. Springer New York, New York, NY. https://doi.org/10.1007/978-1-4614-6849-3

Kuhn, M., Weston, S., Keefer, C., Coulter, N., code), R.Q. (Author of imported C., code), R.R.P.L. (Copyright holder of imported C., 2018. Cubist: Rule- And Instance-Based Regression Modeling.

Louhaichi, M., Borman, M.M., Johnson, D.E., 2001. Spatially Located Platform and Aerial Photography for Documentation of Grazing Impacts on Wheat. Geocarto International 16, 65–70. https://doi.org/10.1080/10106040108542184

Mahlein, A.-K., Rumpf, T., Welke, P., Dehne, H.-W., Plümer, L., Steiner, U., Oerke, E.-C., 2013. Development of spectral indices for detecting and identifying plant diseases. Remote Sensing of Environment 128, 21–30. https://doi.org/10.1016/j.rse.2012.09.019

Merzlyak, M.N., Gitelson, A.A., Chivkunova, O.B., Rakitin, V.Y., 1999. Non‐destructive optical detection of pigment changes during leaf senescence and fruit ripening. Physiologia Plantarum 106, 135–141. https://doi.org/10.1034/j.1399-3054.1999.106119.x

Mevik, B.-H., Wehrens, R., Liland, K.H., Hiemstra, P., 2018. pls: Partial Least Squares and Principal Component Regression.

Mistele, B., Schmidhalter, U., 2010. Tractor-Based Quadrilateral Spectral Reflectance Measurements to Detect Biomass and Total Aerial Nitrogen in Winter Wheat. Agronomy Journal 102, 499–506. https://doi.org/10.2134/agronj2009.0282

Mistele, B., Schmidhalter, U., n.d. A COMPARISON OF SPECTRAL REFLECTANCE AND LASER- INDUCED CHOLORPHYLL FLUORESCENCE MEASUREMENTS TO DETECT DIFFERENCES IN AERIAL DRY WEIGHT AND NITROGEN UPDATE OF WHEAT 14.

Nagler, P.L., Daughtry, C.S.T., Goward, S.N., 2000. Plant Litter and Soil Reflectance. Remote Sensing of Environment 71, 207–215. https://doi.org/10.1016/S0034-4257(99)00082-6

Øvergaard, S.I., Isaksson, T., Korsaeth, A., 2013. Prediction of Wheat Yield and Protein Using Remote Sensors on Plots—Part I: Assessing near Infrared Model Robustness for Year and Site Variations. Journal of Near Infrared Spectroscopy. https://doi.org/10.1255/jnirs.1042

Penuelas, J., Baret, F., Filella, I., 1995. Semiempirical Indexes to Assess Carotenoids Chlorophyll-a Ratio from Leaf Spectral Reflectance. Photosynthetica 31, 221–230.

Peñuelas, J., Filella, I., Biel, C., Serrano, L., Savé, R., 1993. The reflectance at the 950–970 nm region as an indicator of plant water status. International Journal of Remote Sensing 14, 1887–1905. https://doi.org/10.1080/01431169308954010

Peñuelas, J., Gamon, J.A., Fredeen, A.L., Merino, J., Field, C.B., 1994. Reflectance indices associated with physiological changes in nitrogen- and water-limited sunflower leaves. Remote Sensing of Environment 48, 135–146. https://doi.org/10.1016/0034-4257(94)90136-8

Penuelas, J., Pinol, J., Ogaya, R., Filella, I., 1997. Estimation of plant water concentration by the reflectance Water Index WI (R900/R970). International Journal of Remote Sensing 18, 2869–2875. https://doi.org/10.1080/014311697217396

Pimstein, A., Eitel, J.U.H., Long, D.S., Mufradi, I., Karnieli, A., Bonfil, D.J., 2009. A spectral index to monitor the head-emergence of wheat in semi-arid conditions. Field Crops Research 111, 218–225. https://doi.org/10.1016/j.fcr.2008.12.009

Pu, R., 2011. Detecting and Mapping Invasive Plant Species by Using Hyperspectral Data 447–466. https://doi.org/10.1201/b11222-27

Pu, R., Ge, S., Kelly, N.M., Gong, P., 2003. Spectral absorption features as indicators of water status in coast live oak ( Quercus agrifolia ) leaves. International Journal of Remote Sensing 24, 1799–1810. https://doi.org/10.1080/01431160210155965

Qi, J., Chehbouni, A., Huete, A.R., Kerr, Y.H., Sorooshian, S., 1994. A modified soil adjusted vegetation index. Remote Sensing of Environment 48, 119–126. https://doi.org/10.1016/0034-4257(94)90134-1

Qi, J., Marsett, R., Heilman, P., Bieden-bender, S., Moran, S., Goodrich, D., Weltz, M., 2002. RANGES improves satellite-based information and land cover assessments in southwest United States. Eos, Transactions American Geophysical Union 83, 601–606. https://doi.org/10.1029/2002EO000411

Quinlan, 1992. Learning with continuous classes. Proceedings of Australian Joint Conference on Artificial Intelligence 343–348.

Ramirez-Lopez, L., Stevens, A., 2014. prospectr: Miscellaneous functions for processing and sample selection of vis-NIR diffuse reflectance data.

Rao, N.R., Garg, P.K., Ghosh, S.K., Dadhwal, V.K., 2008. Estimation of leaf total chlorophyll and nitrogen concentrations using hyperspectral satellite imagery. The Journal of Agricultural Science 146, 65–75. https://doi.org/10.1017/S0021859607007514

Reyniers, M., Walvoort, D.J.J., Baardemaaker, J.D., 2006. A linear model to predict with a multi‐spectral radiometer the amount of nitrogen in winter wheat. International Journal of Remote Sensing 27, 4159–4179. https://doi.org/10.1080/01431160600791650

Rondeaux, G., Steven, M., Baret, F., 1996. Optimization of soil-adjusted vegetation indices. Remote Sensing of Environment 55, 95–107. https://doi.org/10.1016/0034-4257(95)00186-7

Rouse, J.W., Jr., Haas, R.H., Schell, J.A., Deering, D.W., 1974. Monitoring Vegetation Systems in the Great Plains with Erts. NASA Special Publication 351, 309.

Savitzky, A., Golay, M.J.E., 1964. Smoothing and Differentiation of Data by Simplified Least Squares Procedures. Analytical Chemistry 36, 1627–1639. https://doi.org/10.1021/ac60214a047

Schlemmer, M.R., Francis, D.D., Shanahan, J.F., Schepers, J.S., 2005. Remotely Measuring Chlorophyll Content in Corn Leaves with Differing Nitrogen Levels and Relative Water Content. Agronomy Journal 97, 106–112. https://doi.org/10.2134/agronj2005.0106

Seelig, H.-D., Hoehn, A., Stodieck, L.S., Klaus, D.M., III, W.W.A., Emery, W.J., 2008. The assessment of leaf water content using leaf reflectance ratios in the visible, near‐, and short‐wave‐infrared. International Journal of Remote Sensing 29, 3701–3713. https://doi.org/10.1080/01431160701772500

Serbin, G., Daughtry, C.S.T., Hunt, E.R., Brown, D.J., McCarty, G.W., 2009. Effect of Soil Spectral Properties on Remote Sensing of Crop Residue Cover. Soil Sci. Soc. Am. J. 73, 1545–1558. https://doi.org/10.2136/sssaj2008.0311

Serrano, L., Peñuelas, J., Ustin, S.L., 2002. Remote sensing of nitrogen and lignin in Mediterranean vegetation from AVIRIS data: Decomposing biochemical from structural signals. Remote Sensing of Environment 81, 355–364. https://doi.org/10.1016/S0034-4257(02)00011-1

Sims, D.A., Gamon, J.A., 2002. Relationships between leaf pigment content and spectral reflectance across a wide range of species, leaf structures and developmental stages. Remote Sensing of Environment 81, 337–354. https://doi.org/10.1016/S0034-4257(02)00010-X

Stuckens, J., Swennen, R.L., Coppin, P., Dzikiti, S., Verreynne, S., Verstraeten, W.W., 2011. EXTRACTING PHYSIOLOGICAL INFO FROM A HYPERSPECTRAL TIME SERIES OF A CITRUS ORCHARD. Acta Horticulturae 11–18. https://doi.org/10.17660/ActaHortic.2011.919.1

Takebe, M., Yoneyama, T., Inada, K., Murakami, T., 1990. Spectral reflectance ratio of rice canopy for estimating crop nitrogen status. Plant Soil 122, 295–297. https://doi.org/10.1007/BF02851988

Tucker, C.J., 1979. Red and photographic infrared linear combinations for monitoring vegetation. Remote Sensing of Environment 8, 127–150. https://doi.org/10.1016/0034-4257(79)90013-0

Vogelmann, J.E., Rock, B.N., Moss, D.M., 1993. Red edge spectral measurements from sugar maple leaves. International Journal of Remote Sensing 14, 1563–1575. https://doi.org/10.1080/01431169308953986

Wold, S., Sjöström, M., Eriksson, L., 2001. PLS-regression: a basic tool of chemometrics. Chemometrics and Intelligent Laboratory Systems 58, 109–130. https://doi.org/10.1016/S0169-7439(01)00155-1

Wright, M.N., Ziegler, A., 2017. ranger: A Fast Implementation of Random Forests for High Dimensional Data in C++ and R. Journal of Statistical Software 77. https://doi.org/10.18637/jss.v077.i01

Wu, C., Niu, Z., Tang, Q., Huang, W., 2008. Estimating chlorophyll content from hyperspectral vegetation indices: Modeling and validation. Agricultural and Forest Meteorology 148, 1230–1241. https://doi.org/10.1016/j.agrformet.2008.03.005

Xue, L., Cao, W., Luo, W., Dai, T., Zhu, Y., 2004. Monitoring Leaf Nitrogen Status in Rice with Canopy Spectral Reflectance. Agronomy Journal 96, 135. https://doi.org/10.2134/agronj2004.0135

Yu, K., Lenz-Wiedemann, V., Chen, X., Bareth, G., 2014. Estimating leaf chlorophyll of barley at different growth stages using spectral indices to reduce soil background and canopy structure effects. ISPRS Journal of Photogrammetry and Remote Sensing 97, 58–77. https://doi.org/10.1016/j.isprsjprs.2014.08.005

Zarco-Tejada, P.J., González-Dugo, V., Williams, L.E., Suárez, L., Berni, J.A.J., Goldhamer, D., Fereres, E., 2013. A PRI-based water stress index combining structural and chlorophyll effects: Assessment using diurnal narrow-band airborne imagery and the CWSI thermal index. Remote Sensing of Environment 138, 38–50. https://doi.org/10.1016/j.rse.2013.07.024

Zarco-Tejada, P.J., Rueda, C.A., Ustin, S.L., 2003. Water content estimation in vegetation with MODIS reflectance data and model inversion methods. Remote Sensing of Environment 85, 109–124. https://doi.org/10.1016/S0034-4257(02)00197-9
